# Association of Health Care Work With Anxiety and Depression During the COVID-19 Pandemic: Structural Topic Modeling Study

**DOI:** 10.2196/47223

**Published:** 2023-10-24

**Authors:** Matteo Malgaroli, Emily Tseng, Thomas D Hull, Emma Jennings, Tanzeem K Choudhury, Naomi M Simon

**Affiliations:** 1 Department of Psychiatry Grossman School of Medicine New York University New York, NY United States; 2 Ann S Bowers College of Computing and Information Science Cornell University Ithaca, NY United States; 3 Research and Development Talkspace New York, NY United States

**Keywords:** depression, anxiety, health care workers, COVID-19, natural language processing, topic modeling, stressor, mental health, treatment, psychotherapy, digital health

## Abstract

**Background:**

Stressors for health care workers (HCWs) during the COVID-19 pandemic have been manifold, with high levels of depression and anxiety alongside gaps in care. Identifying the factors most tied to HCWs’ psychological challenges is crucial to addressing HCWs’ mental health needs effectively, now and for future large-scale events.

**Objective:**

In this study, we used natural language processing methods to examine deidentified psychotherapy transcripts from telemedicine treatment during the initial wave of COVID-19 in the United States. Psychotherapy was delivered by licensed therapists while HCWs were managing increased clinical demands and elevated hospitalization rates, in addition to population-level social distancing measures and infection risks. Our goal was to identify specific concerns emerging in treatment for HCWs and to compare differences with matched non-HCW patients from the general population.

**Methods:**

We conducted a case-control study with a sample of 820 HCWs and 820 non-HCW matched controls who received digitally delivered psychotherapy in 49 US states in the spring of 2020 during the first US wave of the COVID-19 pandemic. Depression was measured during the initial assessment using the Patient Health Questionnaire-9, and anxiety was measured using the General Anxiety Disorder-7 questionnaire. Structural topic models (STMs) were used to determine treatment topics from deidentified transcripts from the first 3 weeks of treatment. STM effect estimators were also used to examine topic prevalence in patients with moderate to severe anxiety and depression.

**Results:**

The median treatment enrollment date was April 15, 2020 (IQR March 31 to April 27, 2020) for HCWs and April 19, 2020 (IQR April 5 to April 27, 2020) for matched controls. STM analysis of deidentified transcripts identified 4 treatment topics centered on health care and 5 on mental health for HCWs. For controls, 3 STM topics on pandemic-related disruptions and 5 on mental health were identified. Several STM treatment topics were significantly associated with moderate to severe anxiety and depression, including working on the hospital unit (topic prevalence 0.035, 95% CI 0.022-0.048; *P*<.001), mood disturbances (prevalence 0.014, 95% CI 0.002-0.026; *P*=.03), and sleep disturbances (prevalence 0.016, 95% CI 0.002-0.030; *P*=.02). No significant associations emerged between pandemic-related topics and moderate to severe anxiety and depression for non-HCW controls.

**Conclusions:**

The study provides large-scale quantitative evidence that during the initial wave of the COVID-19 pandemic, HCWs faced unique work-related challenges and stressors associated with anxiety and depression, which required dedicated treatment efforts. The study further demonstrates how natural language processing methods have the potential to surface clinically relevant markers of distress while preserving patient privacy.

## Introduction

During the COVID-19 pandemic, health care workers (HCWs) faced mounting stress as they cared for patients experiencing a disease that, to date, has infected 538 million globally and 85 million in the United States alone [1]. Surges in US infection rates forced hospitals to operate at greater than 100% capacity [2], with COVID-19 hospitalizations in 18 states exceeding 10% of all available beds and 7 states operating at more than 15% overcapacity [3]. As a result, HCWs faced overwhelming workloads, longer hours, increased personal infection risk, equipment shortages, sleep disruption, and at times the need to make ethically challenging decisions, such as rationing care for patients [4-8]. This increased burden on HCWs was further aggravated by the loss of social support due to quarantine policies and the fear of infecting family and friends [6,7,9].

The well-being of HCWs is the foundation of a well-functioning health care system [10-12]. Prior to the pandemic, HCWs already faced higher rates of anxiety, depression [13], and suicidal ideation [14] compared to the general population [13,15]. The sudden increase in professional and personal stress due to COVID-19 put HCWs, an already vulnerable population with barriers to treatment access [16], at further risk for developing symptoms of anxiety and depression [5,6,9]. Prior studies have linked depression and anxiety in HCWs to decreased patient safety and increased medical errors [17-20]. Given the adverse consequences of psychological stress for HCWs and their patients, it is crucial to better understand the core concerns associated with mental health symptoms such as anxiety and depression in HCWs, especially during periods of acute stress like COVID-19 surges. It is especially crucial to study these concerns in ways that preserve the privacy and anonymity of HCWs, given the professional stigma reported by some health care providers who seek mental health treatment [21-23].

Hastened by the pandemic, recent advances have been made in developing and disseminating digital mental health interventions to address acute and long-term treatment barriers, including mobile apps and telehealth platforms connecting patients to mental health providers [24]. Such interventions offer a unique opportunity for understanding and addressing the mental health concerns of vulnerable populations like HCWs. Despite their potential, little research has examined the adoption of digital health treatment by HCWs during COVID-19.

In addition to providing flexible options for clinical engagement, digital treatment delivery enables the automatic collection of large amounts of treatment data, which in turn can be analyzed in an aggregated and deidentified fashion using machine learning (ML) methods. Researchers in digital psychiatry and ubiquitous computing have used ML to develop passive measures for mental health concerns, which can be refined into clinically relevant markers for symptom severity and embedded into treatment pathways [25-27]. ML-based natural language processing (NLP) holds particular promise for the study of mental health concerns, as it allows the study of verbal expressions of distress at scale, capturing clinically relevant linguistic features from unstructured text as the patient-therapist interaction unfolds [28]. Of particular interest in the study of psychotherapy transcripts is topic modeling, an unsupervised NLP method to parse semantic structures (or topics) from large corpora of text without the need for line-by-line annotation [29]. Topic modeling has been used to generate knowledge in multiple areas of science [30], and previous uses of topic modeling in mental health include the detection of depression [31] and anxiety [32], also in the context of the COVID-19 pandemic [33]. In brief, topic modeling imagines that every document within a corpus contains a mixture of corpus-wide distributions of words within a fixed vocabulary. Topic modeling algorithms seek to find the topics that best characterize a given corpus across documents, as a means to understand the core content of potentially difficult texts (such as therapy transcripts) at scale [28]. Structural topic models (STMs) also enable the study of covariates in their influence on topic prevalence, or the proportion of a document associated with a topic, and topical content, or the distribution of words used within a topic. Compared to lexicon-based methods, topic modeling allows assessing context-specific language (such as medical terminology) within the corpus of transcripts. Compared to embeddings, which capture semantic similarity at the word or sentence level, topic modeling can also uncover broader thematic associations across transcripts, to then group individuals based on topical themes emerging from the transcripts. For collections of texts like transcripts of psychotherapy sessions, topic modeling also offers the potential to be more privacy preserving: topic models process text into distributions of keywords and enable researchers to study the semantic content of sensitive therapy transcripts while preserving treatment-seekers’ privacy by minimizing exposure of personally identifiable or sensitive data. Topic modeling can provide empirical insights into the stressors experienced by medical providers during this highly stressful period of the pandemic. Moreover, by linking specific concerns identified via topic modeling with depression and anxiety symptoms measured with validated scales, linguistic features can be developed to serve as passive computational markers [34] of distress, with the potential to highlight areas of risk or need for clinical attention. Identifying the most disruptive risk factors for HCWs would support improvements in treatment planning and inform the selection of mental health resources for HCWs now, during future COVID-19 waves, or other widespread epidemics.

**Figure 1:**
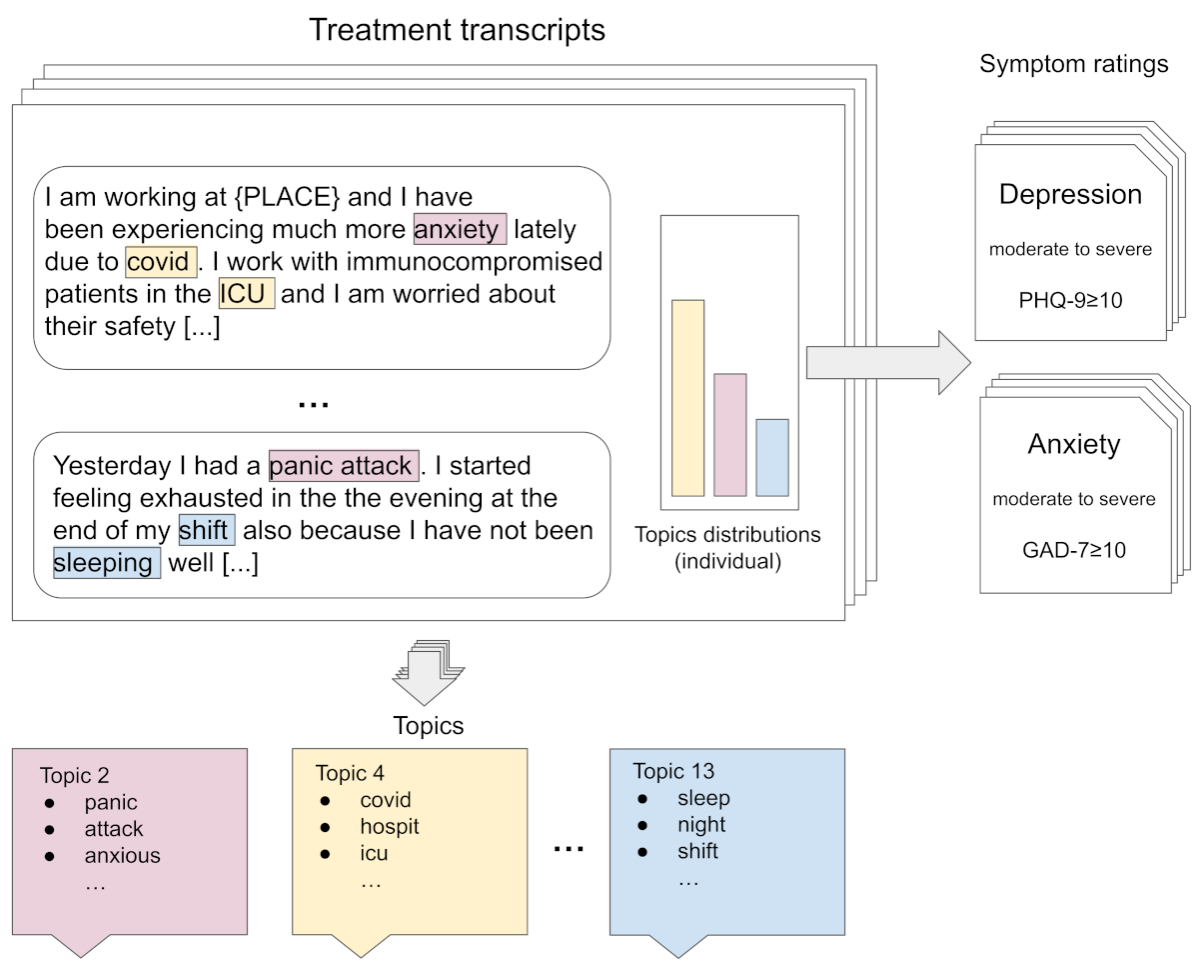
Schematic overview of topic modeling with fictitious example of a transcript. Topics are generated across the full transcript corpora. Individual topic distributions are then associated with their respective symptom ratings. GAD-7: General Anxiety Disorder Scale-7; ICU: intensive care unit; PHQ-9: patient health questionnaire-9.

In this study, we examined deidentified treatment transcripts from 820 HCWs and 820 matched controls who received digitally delivered psychotherapy from licensed therapists during the first US surge of the COVID-19 pandemic, between March and July 2020. Our aim was to identify the unique treatment needs of HCWs characterized as treatment topics compared to non-HCW matched controls. We analyzed transcripts using STM to assess the topic in the deidentified transcripts and their associations with symptom levels. Specifically, we used topic modeling to analyze therapeutic conversations during the first 3 weeks of treatment in HCW and controls and identified emerging topics in a privacy-preserving fashion. We also assessed the association of these topics with moderate to severe levels of anxiety and depression (Figure 1).

## Methods

### Participants and Setting

Our sample consisted of self-referred HCWs from the United States seeking digitally delivered psychotherapy in spring 2020, amidst the first US surge of COVID-19 hospitalizations. HCWs were defined as health care and medical providers (eg, physicians, nurses, residents, emergency medical service providers, and social workers) with an active National Provider Identifier (NPI) profile at the time of treatment. Services were donated by a telehealth platform [35] as part of an initiative to provide 1 month of free treatment to essential HCWs. Eligibility was verified by the platform through employment and NPI verification. In order to distinguish health care–specific and general population stressors related to COVID-19, we included a matched control sample of non-HCWs from the general population seeking the same treatment service as the HCW sample in spring 2020. Non-HCW patients accessed the platform through employee assistance programs, self-referral, and as benefits through individual insurance. From this outpatient pool, a control sample was matched to HCWs based on demographics, symptom scores, US state of residency, and treatment start date. Control matching was performed algorithmically, and matching procedures are described in Multimedia Appendix 1 [5,29,36-49].

Before starting treatment, HCWs and controls received a primary ICD-10 diagnosis based on a standardized intake evaluation by a licensed clinician to identify presenting complaints and treatment history. Following the intake, HCWs and controls were matched to a licensed therapist and received psychotherapy through messages exchanged using a HIPAA (Health Insurance Portability and Accountability Act)-compliant interface for smartphones and computers. The inclusion criteria were (1) living in the United States, (2) being an English speaker, and (3) having regular internet or cellphone access (to access the digitally delivered treatment). Exclusion criteria for both samples were (1) any condition deemed by the intake clinician to require hospitalization; (2) suicidal thoughts or behavior sufficient to be marked a yes on any of questions 3 through 6 (at least thoughts about a potential suicide method) on the Columbia Suicide Severity Rating Scale Lifetime-Recent [36]; (3) current or past diagnoses of bipolar disorder, substance use disorders, schizophrenia spectrum disorders, or psychotic disorders; (4) patients who did not have complete baseline symptom measures; and (5) patients who did not have treatment transcripts available. Last, as exclusion criterion 6, during matching procedures, we excluded from the control group any health care professional. An overview and schematic of the sampling procedure in this study are reported in Multimedia Appendix 1 [5,29,36-49].

The final sample consisted of 820 HCWs and 820 matched controls. The median treatment start date for HCWs was April 15, 2020 (IQR March 31 to April 27, 2020). For the matched control group, the median treatment start date was April 19, 2020 (IQR April 5 to April 27, 2020).

### Data Sources and Measures

#### Transcripts

Psychotherapy treatment transcripts consisted of deidentified messages between patients and their therapists with their corresponding timestamp (ie, date and time of delivery) in masked form for the author role of the text. All transcript data were deidentified using an algorithm to scrub out any personal identifiers, proper nouns, locations, dates, and other potential identifiers. Transcripts were truncated to include only messages sent by patients from the initial intake to their first outcome survey, typically 3 weeks after treatment initiation. HCWs and control transcripts were both preprocessed for analysis: numbers, punctuation, stopwords, and anonymization terms (eg, “{NAME}”) were removed; the remaining words were stemmed and converted to their root form (eg, *computing* was changed to *comput*). The “vocabulary” of unique words across the preprocessed transcripts was then made more tractable by removing words that occurred in less than 50 documents and then removing documents that contained no words. The final HCW corpus contained 820 therapy transcripts and a vocabulary of 1208 unique terms across 225,219 tokens. The final control corpus contained 820 transcripts and a vocabulary of 1259 unique terms across 217,321 tokens.

#### HCW Occupations

NPI information for HCWs in our study was not available as data due to privacy reasons. To assess the distribution of specific health care professions in the HCW sample anonymously, we developed a heuristic classification algorithm. The algorithm detected instances in the transcripts where patients self-identified as HCWs or spoke about their professional roles. Code, heuristics, and accuracy metrics of the heuristic classification algorithm are further reported in Multimedia Appendices 1 [5,29,36-49] and 3.

#### Psychiatric Symptom Measures

Depression symptoms were measured at the beginning of treatment using the Patient Health Questionnaire-9 (PHQ-9) [50], and anxiety symptoms were measured using the General Anxiety Disorder Scale-7 (GAD-7) [51]. The PHQ-9 assesses for depressive symptoms over the past 2 weeks on a 4-point Likert scale (0=“not at all” to 3=“nearly every day”), with a total maximum score of 27. The GAD-7 examines symptoms of anxiety over the past 2 weeks on a 4-point Likert scale (0=“not at all” to 3=“nearly every day”), with a total maximum score of 21. A score of 10 or more on the PHQ-9 identifies the presence of clinically significant moderate-to-severe depression [50]; a score of 10 or more on the GAD-7 identifies the presence of clinically significant moderate-to-severe anxiety [51].

### Data Analysis

#### Treatment Topic Identification

All analyses were conducted in Python (version 3.9.9) and in R (version 4.1.2) [37], using the package *stm* [38] for topic modeling. Additional model specifications, diagnostic analyses, model selection procedures, and code for all analyses are reported in Multimedia Appendices 1 [5,29,36-49] and 2.

STMs were used to identify topics in the HCW and matched control corpora. We used a mixed statistical and human validation process to select topics (K=30) for analysis in both HCW and control data sets (see Multimedia Appendix 1 [5,29,36-49] for full details). After identifying these topics, results from the STM were manually coded to characterize their relevance to one of three areas of interest: (1) mental health, (2) COVID-19 pandemic-related disruptions, and (3) health care. Classification of relevant topics was determined through the consensus of a panel of experts consisting of 2 doctoral-level clinical psychologists (MM and TDH), 1 psychiatrist (NMS), and 1 NLP researcher (ET). Topics were examined to understand their content based on their most characteristic words, determined using the harmonic mean of word frequency and exclusivity across topics [39].

#### Topics and Clinical Levels of Depression and Anxiety

We used STM effect estimators to study the association between topics discussed by each patient with moderate to severe depression or anxiety. Specifically, we ran logistic-normal generalized linear models examining the association between the prevalence of relevant topics in a patient’s transcripts and their binarized psychopathology score, with GAD-7 or PHQ-9 symptom scores ≥10 classed as moderate-to-severe anxiety or depression (Figure 1). The study combined PHQ-9 ≥10 or GAD-7 ≥10 cutoffs to account for the high prevalence of anxiety and depression comorbidity [52]. To estimate the parameters of the generalized linear models, we used a global approximation to the average covariance matrix governing the variational posterior (vs a per-document approximation that was less computationally tractable). Topic prevalence for a particular topic is contrasted for 2 groups within a categorical covariate (none-to-mild vs moderate-to-severe symptoms). For each data set, all topics (K=30) were modeled and reported in Multimedia Appendix 1 [5,29,36-49]; here, we report only those topics manually characterized as relevant.

### Ethics Approval

All patients and clinicians gave informed consent to use their data in a deidentified, aggregated format for research purposes as part of the user agreement before they began using the platform. Study procedures were approved by the Cornell University Institutional Review Board (2004009578).

## Results

### Sample Characteristics

**Figure 2:**
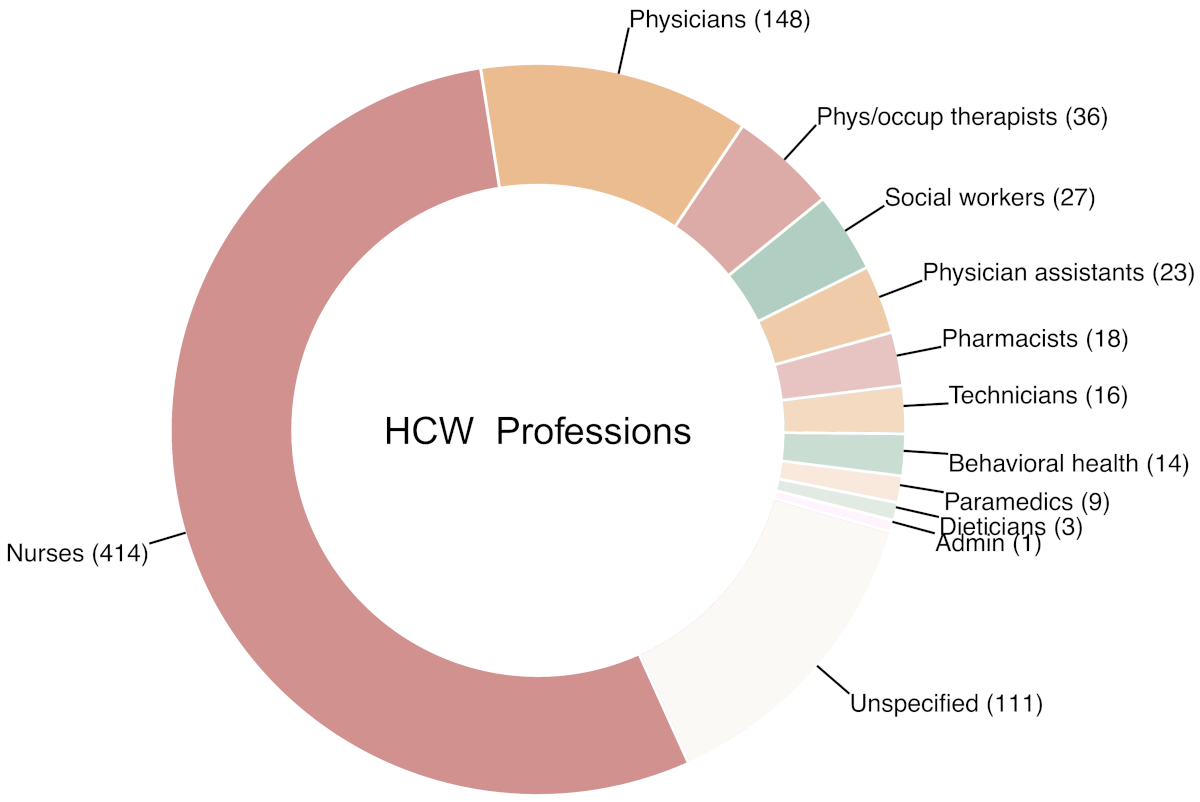
Algorithmically identified distribution of medical professions in the HCW sample (n=820). HCW: health care worker.

HCW patients (n=820, Table 1) modally identified as female (746/820, 91%). The mean age in the sample was 31.3 (SD 5.7) years. They were distributed across the United States, with the largest concentrations in New York State (114/820, 13.1%) and California (107/820, 13%). Figure 2 reports the distribution of professions in the HCWs identified from the transcripts, with nurses (414/820, 50.5%) and physicians (148/820, 18.1%) being the most frequent health care occupations. For 289 HCWs (35.2%), this was reportedly the first psychotherapy treatment experience. Primary diagnoses given by intake clinicians for HCWs included anxiety disorders (463/820, 56.5%), of which 100 were generalized anxiety disorders (12.2%). Trauma- and stressor-related disorders (275/820, 35.5%) were next most common, with adjustment disorders as the modal diagnosis (219/820, 26.7%) in this category. Finally, depressive disorders (67/820, 8.2%) were least common and included 45 diagnosed with major depressive disorder (5.5%). Based on PHQ-9 and GAD-7 cutoffs, the prevalence of moderate to severe depression in the HCW sample at the beginning of treatment was 43.9% (n=360) and moderate to severe anxiety was 68.5% (n=562) A total of 601 (73.3%) HCWs had either moderate to severe anxiety or depression at baseline. In the matched control sample, 560 (68.3%) had moderate to severe anxiety and 408 (49.8%) had moderate to severe depression, with 601 (73.3%) having either moderate to severe anxiety or depression at baseline. Characteristics for the sample of matched controls (n=820) are reported in Table 1.

**Table 1:** Demographic and clinical characteristics of health care worker sample (n=820) and matched control sample (n=820).

Variable		Health care workers	Matched controls
Age (years), mean (SD)		31.3 (5.7)	32 (6.6)
**Diagnosis, n (%)**			
	Anxiety disorders	463 (56.5)	429 (52.3)
	Trauma- and stressor-related disorders	275 (35.5)	137 (16.7)
	Depressive disorders	67 (8.2)	225 (27.4)
	Other disorders	15 (1.8)	29 (3.5)
**Gender, n (%)**			
	Female	746 (91)	682 (83.2)
	Male	69 (8.4)	125 (15.2)
	Other	5 (0.6)	13 (1.6)
**State, n (%)**			
	California	114 (13.1)	132 (16.1)
	New York	107 (13)	108 (13.2)
	Florida	55 (6.7)	56 (6.8)
	Texas	48 (5.9)	38 (4.6)
	Illinois	45 (5.5)	33 (4)
	Massachusetts	41 (5)	34 (4.2)
	Pennsylvania	35 (4.3)	34 (4.2)
	North Carolina	30 (3.7)	28 (3.4)
	New Jersey	30 (3.7)	30 (3.7)
	Washington	28 (3.4)	27 (3.9)
	Other US states	287 (35)	300 (36.6)
**Anxiety**			
	GAD-7^a^ score, mean (SD)	12.4 (4.9)	12.3 (5.1)
	Moderate to severe (GAD-7 ≥10), n (%)	562 (68.5)	560 (68.3)
**Depression**			
	PHQ-9^b^ score, mean (SD)	9.4 (5.7)	10 (5.8)
	Moderate to severe (PHQ-9≥10), n (%)	360 (43.9)	408 (49.8)
**Treatment**			
	First experience (Yes), n (%)	289 (35.2)	272 (33.2)
	Start date (month/day/year), median (IQR)	04/15/2020 (03/31/2020-04/27/2020)	04/19/2020 (4/5/2020-4/27/2020)

^a^GAD-7: General Anxiety Disorder Scale-7.

^b^PHQ-9: Patient Health Questionnaire-9.

### Treatment Topics

**Figure 3:**
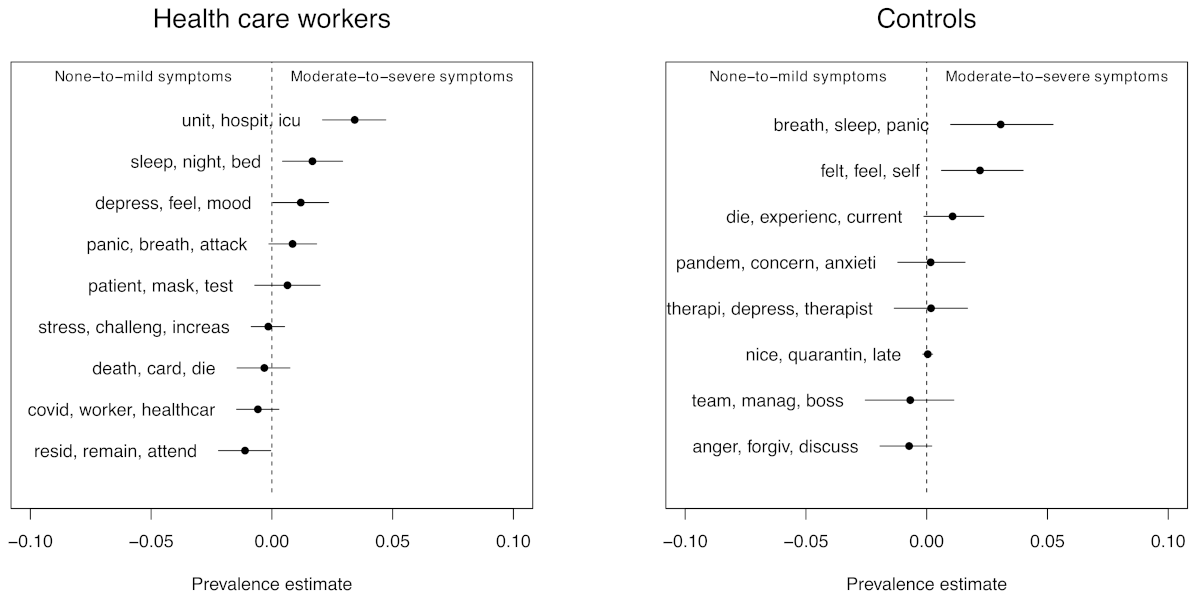
Structural topic model estimates of association between psychiatric symptoms and mean topic prevalence. Topics on the right side of the dotted line have higher prevalence in Controls and Health care Workers with moderate to severe anxiety and depression.

STM of psychotherapy transcripts identified 30 conversational themes for HCWs and 30 topics for non-HCW controls. Inspection of the topics showed a cluster of themes relevant to mental health and a cluster relevant to health care and the pandemic (Table 2). All topics emerging from the transcripts are reported in Figure 3.

HCWs discussed 4 topics related to practicing medicine. Examination of the most frequent words exclusive to HCWs indicated treatment topics focused on (1) virus-related fears (topic H3: *covid*, *worker*, *healthcar*), (2) working on the hospital floor and intensive care units (H4: *unit*, *hospit*, *icu*), (3) patients and masks (H16: *patient*, *mask*, *test*), and (4) health care roles including resident and attending (H29: *resid*, *remain*, *attend*). In contrast, therapy transcripts from controls contained only 1 topic about the COVID-19 pandemic (C25: *pandem*, *concern*, *anxiety*) and 1 occupational-related topic (C27: *team*, *manag*, *boss*).

HCWs and controls each discussed 5 topics with their therapist related to their mental health, endorsing panic attacks (HCW H2: *panic*, *breath*, *attack*; control C21: *breath*, *sleep*, *panic*), affective disturbances (HCW H15: *depress*, *feel*, *mood*; control C16: *felt*, *feel*, *self*), and grief (HCW H30: *death*, *card*, *die*; control C19: *die*, *experienc*, *current*). HCWs also endorsed sleep disturbances (H13: *sleep*, *night*, *bed*), and stress (H21: *stress*, *challeng*, *increase*). Among health care and mental health topics, HCWs most frequently discussed sleep disturbances (H13: *sleep*, *night*, *bed*) and the hospital floor (H4: *unit*, *hospit*, *icu*). Multimedia Appendix 1 [5,29,36-49] reports the proportions of all topics in the HCWs and control transcripts.

**Table 2:** Psychotherapy topics referencing mental health, health care, and COVID-19 in health care workers and matched controls.

Sample, category, and topic				Top 10 terms (frequency and exclusivity)^a^
**Health care workers (n=820)**				
	**Health care**			
		H3	covid, worker, healthcar, hospit, patient, physician, current, week, promot, doctor	
		H4	unit, hospit, icu, nurs, virus, news, sick, covid, safe, fear	
		H16	patient, mask, test, shift, unit, wear, staff, icu, ppe, coronavirus	
		H29	resid, remain, attend, program, becom, answer, clinic, mayb, mean, studi	
	**Mental health**			
		H2	panic, breath, attack, symptom, anxious, anxieti, exercis, chest, tool, calm	
		H13	sleep, night, bed, shift, asleep, wake, usual, fall, morn, relax	
		H15	depress, feel, mood, anyth, suicid, quarantin, sad, episod, sometim, hard	
		H21	stress, challeng, increas, relief, level, team, stressor, overal, focus, line	
		H30	death, card, die, grief, code, credit, pass, deal, charg, enter	
**Matched controls (n=820)**				
	**Pandemic disruptions**			
		C25	pandem, concern, anxieti, situat, cope, corona, group, relat, social, extrem	
		C11	nice, quarantin, late, gym, enjoy, crazi, glad, weather, excit, heavi	
		C27	team, manag, boss, project, task, routin, offic, work, cowork, hour	
	**Mental health**			
		C21	breath, sleep, panic, sick, attack, night, anxious, anxieti, worri, calm	
		C16	felt, feel, self, negat, anxious, thought, sad, bad, scare, boyfriend	
		C9	therapi, depress, therapist, issu, anxieti, disord, eat, month, cost, coupl	
		C2	anger, forgiv, discuss, hurt, angri, behavior, intak, lie, said, sexual	
		C19	die, experienc, current, attack, medic, alcohol, rate, daili, health, panic	

^a^Most frequent and exclusive words that distinguish each topic in patients’ transcripts.

### Topics and Clinical Levels of Depression and Anxiety

After determining the distribution of topics emerging from treatment transcripts, we examined the association of topics discussed in psychotherapy with patients’ moderate-to-severe anxiety or depression at 3 weeks of treatment. Effect estimates and their 95% CIs are reported in Figure 3. Discussion of the hospital and its locations (H5: *unit*, *hospit*, *icu*) was significantly more prevalent among HCWs with moderate to severe anxiety or depression (topic prevalence=0.035, 95% CI 0.022-0.048; *P*<.001). This effect was not observed in the matched controls for the pandemic-relevant topic (C25, *pandem*, *concern*, *anxiety*: prevalence=0.003, 95% CI –0.012 to 0.018; *P*=.67), nor for the work-relevant topic (C27, *team*, *manag*, *boss*: prevalence=–0.005, 95% CI –0.021 to 0.011; *P*=.55). Other topics were significantly more prevalent for symptomatic HCWs, including endorsing affective disturbances (H15, *depress*, *feel*, *mood*: prevalence=0.014, 95% CI 0.002-0.026; *P*=.03) and sleep disturbances (H13, *sleep*, *night*, *bed*: prevalence=0.016, 95% CI 0.002-0.030; *P*=.02). No other mental health and health care topics occurred at significantly higher frequency in HCWs with moderate to severe anxiety or depression, with weak trends observed for discussions related to panic attacks, grief, and mask-related concerns. Controls with moderate to severe symptoms were more likely to discuss affective disturbances (C16: prevalence=0.021, 95% CI 0.005-0.037; *P*=.01). Numeric estimates for all HCWs and control topics are reported in Multimedia Appendix 1 [5,29,36-49].

## Discussion

### Overview

In this study, we examined topics for 820 HCWs and 820 matched general-population outpatients undergoing psychotherapy through a telehealth platform in spring 2020 during the first US wave of COVID-19 and their associations with moderate to severe depression and anxiety. In total, 3 weeks of treatment transcripts were examined using NLP methods, enabling elucidation of the content of therapy discussions automatically at scale and in a privacy-preserving way. Results indicated significant differences in the proportion of health care–related topics between HCW and control cohorts, as well as their association with moderate to severe anxiety and depression.

Analysis of the distribution of NLP-derived treatment topics indicated that HCWs extensively discussed health care–related topics in psychotherapy. Specifically, HCWs had 4 conversational themes around health care, while controls only had 1. This finding is consistent with the increased work-related stressors experienced by HCWs during the COVID-19 pandemic, when they were particularly vulnerable to work-related adverse impacts compared to the general population, given the increased professional and personal responsibilities they faced. These unique effects of COVID-19 made HCWs specifically vulnerable to mental health problems compared to the general population [53]. In addition to the effect of potential stressors experienced by HCWs during the COVID-19 pandemic, this finding is also consistent with prior literature indicating that work-related stress is almost twice as prevalent for HCWs as for workers in other fields after controlling for work hours, with physicians at the front line of care at greatest risk [54]. Unique factors that contribute to this include longer hours and greater difficulty with work-life integration compared to other US workers.

Analysis of the prevalence of topics and their association with symptomatology indicated that among HCWs, discussion of hospital settings was significantly associated with moderate to severe anxiety and depression. This association was unique for HCWs and not present in the general-population outpatients, despite shared anxiety, work, and health-related concerns during the pandemic [55]. Discussion of sleep disturbances and mood difficulties were also significantly associated with moderate to severe anxiety and depression in HCWs. These findings confirm the connection between anxiety, depression, and concerns related to being a practicing medical professional during the COVID-19 pandemic [56]. Although not assessed here, possible underlying contributing factors may be hypothesized to include longer exposures to stressful working environments, a higher level of personal responsibility in critical situations, and increased sleep disruption [19]. Sleep deprivation among HCWs has been consistently linked to increases in anxiety, depression, and suicidal ideation [57]. These findings are especially robust for HCWs who work longer hours, who work night shifts, and who have less time off between their shifts [58]. Existing literature supports a similar relationship for how work-related stress and anxiety and depressive symptoms mutually reinforce each other [59].

### Strengths and Limitations

Findings of this study are unique due to the large corpus of treatment transcripts from HCWs during the initial phase of the COVID-19 pandemic, and data analytic methods exploring the use of computational linguistics to identify stated risk factors. To the growing body of literature documenting the challenges posed to mental health and well-being by the COVID pandemic, we contribute a proof-of-concept demonstrating that web-based therapy platforms can serve as unique observatories for the mental health needs of hard-to-reach populations like HCWs. This study has several limitations. First, our sample consisted of self-referred patients, and differences in access to telehealth services could reduce the generalizability of results. Second, our sample showed a skew toward female individuals and nursing occupations, although this distribution aligned consistently with US population occupational statistics for HCWs [60]. Third, we focused our analysis on a concatenation of all of a patient’s talk turns during the first 3 weeks of treatment. Future work should focus on complex modeling of topics over time, for example using sequential models to examine topics turn-by-turn, as well as models incorporating therapists’ talk turns. Fourth, our findings emerged from the corpus the STM was trained on and might not generalize when applied to different corpora, such as transcripts in languages other than English. Future studies should consider using pretrained large language models on wider corpora of clinical data for more generalizable topic representations across multiple domains and languages [61]. Fifth, topic associations with symptoms were limited to data from validated self-report measures, and other methods to capture psychiatric symptoms may return different results.

### Privacy and Ethics

Important ethical considerations about patient privacy need to be made when accessing sensitive health information such as psychotherapy transcripts. This study included several privacy-preserving measures to reduce risks associated with the study. First, all patients and clinicians gave informed consent to the use of their data in a deidentified and aggregated format for research purposes as part of the user agreement they signed before they began using the platform. All procedures were approved by the university institutional review board. Second, all transcripts were deidentified by the platform prior to the research team accessing the data. Deidentification removed any personal identifiers, like proper nouns, locations, and dates, among other potential identifiers. Third, we limited our analyses to the outputs of STM, which are distributions of common words less likely to reveal private information than the raw text. The first 2 authors (MM and ET) handled the primary analyses and were the only authors to view any portion of raw deidentified text, accessed exclusively as part of model development. Fourth, HCWs’ NPIs and associated information were not accessed as part of the study. Rather, specific health care occupations were identified using named entity recognition on the deidentified transcripts. This solution allowed us to extract occupational information while minimizing access to the raw deidentified transcripts, thus further preserving patient privacy.

### Conclusions

Among US HCWs seeking psychotherapy treatment in spring 2020 during the first wave of the COVID-19 pandemic, discussion of workplace-related concerns was uniquely associated with moderate to severe anxiety and depression. The association between health care work and psychiatric symptoms was unique, going beyond other quality-of-life factors potentially related to work such as poor sleep hygiene. We contribute to the literature on the psychological burden associated with health care work by demonstrating that HCW-specific content related to anxiety and depression emerges naturally in the context of web-based psychotherapy. These findings highlight the unique mental health concerns faced by HCWs during the COVID-19 pandemic, a time with significantly increased work demands, lack of social support, and fear of infection from work activity for HCWs and their families. These stressors were in addition to work-related stressors regularly faced by HCWs [54]. The results of this research could help pinpoint the key factors contributing to the high levels of depression and anxiety among HCWs and fill the gaps in care. The increased stress put on HCWs during COVID-19 along with the established link between HCWs’ mental health and societal well-being supports the critical need to prioritize mental health treatment provision for HCWs.

As mental health risk factors were captured automatically from transcripts using NLP methods, the study also serves as a proof of concept for the automated detection of psychological distress in HCWs. One of the main advantages of NLP markers is that they can identify specific language patterns that are associated with anxiety and depression. Unlike traditional assessment methods, such as self-reported surveys and interviews, NLP markers from psychotherapy platforms present a passive and less burdensome way to assess therapy-seekers’ mental health, akin to the digital biomarkers of mental health researchers have developed from wearable and smartphone data. Defining and validating NLP markers of anxiety and depression could lead to more accurate and reliable assessments, which would be beneficial for both patients and health care providers. Moreover, NLP markers could help to better understand the underlying mechanisms of anxiety and depression by teasing patients into different subgroups based on their specific needs and characteristics. By identifying these patterns, we could tailor treatment and intervention strategies to the specific needs of each patient in clinical settings [62,63]. Eventually, NLP methods could support the advancement of personalized medicine approaches where mental health needs can be estimated routinely using automated methods in ecological or real-world settings. This could be achieved by designing digital apps [64] that offer periodic checks to elicit narrative content about potential risk factors and stressors. Transcripts of the narratives could then be analyzed to extract conversational topics associated with probabilities of experiencing distress through NLP techniques such as STM. For example, this approach could identify language patterns focusing on work-related stressors (such as our HCW sample) or behavioral disturbances (eg, poor sleep hygiene), and then offer personalized triage and resource recommendations. Similar work has been conducted in the context of crisis counseling platforms to analyze patients’ messages for suicidal ideation [65,66]. Offering mental health resources at scale through automated recommendations could help HCWs overcome barriers to treatment access including stigma and unpredictable work hours. Given the high-stress nature of the health care profession, there is vast potential for designing automated systems that can proactively evaluate individual needs and provide personalized resources for preventive care.

## Appendix

Multimedia Appendix 1 Study sample flow chart and details on structural topic modeling.

Multimedia Appendix 2 Analytic code: topic modeling.

Multimedia Appendix 3 Analytic code: job identification algorithm.

## Abbreviations

GAD-7: General Anxiety Disorder Scale-7
HCW: health care worker
ML: machine learning
NLP: natural language processing
NPI: National Provider Identifier
PHQ-9: Patient Health Questionnaire-9
STM: structural topic model
HIPAA: Health Insurance Portability and Accountability Act

## Data Availability

Deidentified patient data may be made available upon completion of a data use agreement and data security review with Talkspace. Analytic code describing natural language processing methods and algorithms is available in Multimedia Appendices 2 and 3.
