# Supplementary material for: Association of Health Care Work With Anxiety and Depression During the COVID-19 Pandemic: Structural Topic Modeling Study
Source: JMIR AI. 2023 Oct 24;2:e47223. doi: 10.2196/47223 (PMC11041488; doi:10.2196/47223)

Supplementary Materials

Matched controls and Recruitment Overview

Supplementary Fig. 1. Study sample flow chart

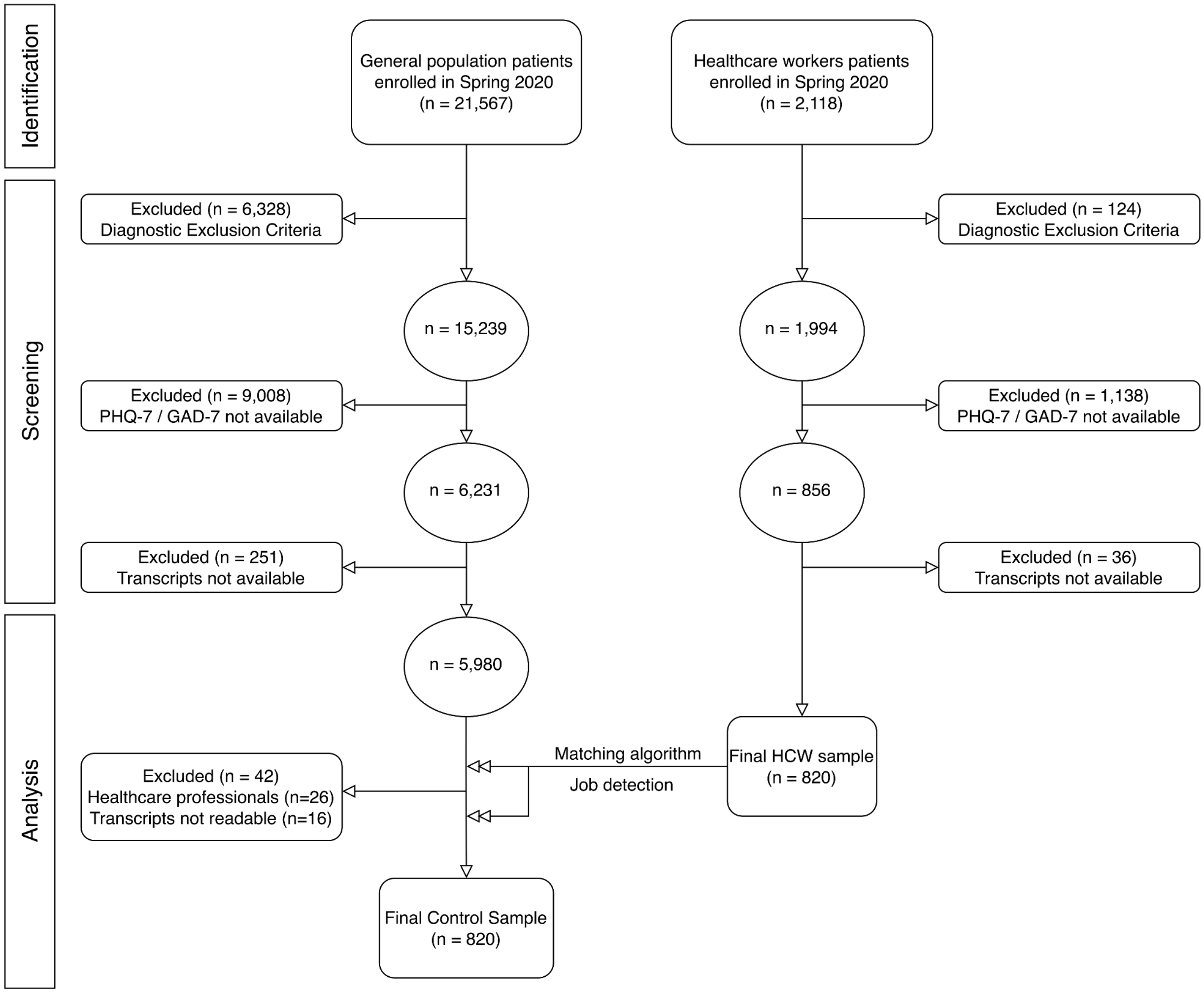

The study recruitment flowchart is reported in Supplementary Figure 1. Our control sample consisted of general population adult outpatients from the United States seeking digitally delivered psychotherapy using the same telehealth platform (www.talkspace.com) as per the healthcare worker (HCW) sample. Compared to HCW who received treatment through a COVID-19 program promotional event, general population patients accessed the platform through employee assistance programs, self-referral, and as a behavioral

health benefit through individual insurances. Procedures to match controls with HCW are as follows: Inclusion criteria consisted of: 1) living in the United States; 2) being an English speaker; 3) having regular Internet or cellphone access. Initial exclusion criteria consisted of: 1) any condition deemed by an intake clinician to require hospitalization (e.g. psychotic episode, life emergencies requiring in-person support, etc); 2) suicidal thoughts and/or behavior sufficient to be marked a "Yes" on any of questions three through six (at least thoughts about a potential suicide method), on the Columbia Suicide Severity Rating Scale Lifetime-Recent [36]. We first identified all patients receiving treatment within the same time frame of the HCW sample (i.e., treatment date ≥ earliest HCW treatment date & treatment date ≤ latest HCW treatment date). This first selection led to an initial subsample of 21,567 patients. We then applied the same additional exclusion criteria as the healthcare worker sample: 3) current or past diagnosis of bipolar disorder, substance use disorders, schizophrenia spectrum, and psychotic disorders; 4) missing both Patient Health Questionnaire-9 (PHQ-9) and General Anxiety Disorder Scale-7 (GAD-7) from treatment baseline; 5) lack of available treatment transcripts. Exclusion criteria further reduced the sample to 5,980 patients for algorithmic treatment matching with the HCW sample. In addition, following initial matching subselection, we 6) excluded any behavioral or mental healthcare worker, based on self-identification or HCW profession mentioned in the treatment transcripts. Healthcare occupations were verified using the job detection algorithm and hand-labeling as per the HCW sample (see "Healthcare profession detection algorithm" supplementary section). Algorithmic matching was then re-run as needed.

Control matching procedures

To identify which of the 5,980 general population outpatients had similar characteristics to the HCW sample we performed algorithmic matching based on nonparametric methods. First, we merged the two samples into a dataset containing the matching parameters of interest (i.e., demographics and clinical characteristics). Second, a binary variable (i.e. group) was created to indicate if the patient was part of the HCW group (n=820) or the larger outpatient sample (n=5,980), and used as the treatment variable for the matching algorithm. Third, we ran the matching procedure to identify the best fitting subset of general population patients to serve as controls. Parameters for the matching consisted of US state of residence PHQ-9 score, and GAD-7 score, start of treatment date, age, and gender. Matching was performed in R[3], version 4.1.2, using the package MatchIt [47]. We used nearest neighbor matching being the most frequently used matching method [49]. Nearest neighbor is a greedy algorithm that examines each individual in the sample of interest (i.e., HCW) and selects the closest eligible control to be paired. Matching distance was determined based on propensity scores estimated using logistic regressions. The matching code is reported below:

```
match.output <- matchit(
```

```
formula = group ~ state + PHQ9 + GAD7 + treatment_date + age + gender,
data = data,
method = "nearest", distance = "glm",
link = "logit", estimand = "ATT",
exact = NULL, mahvars = NULL, antiexact = NULL, discard = "none",
reestimate = FALSE, s.weights = NULL, replace = FALSE,
m.order = NULL,caliper = NULL, std.caliper = TRUE,
ratio = 1, verbose = FALSE, include.obj = FALSE)
```

The first round of matching procedure resulted in 820 controls whose characteristics best matched the HCW set based on demographic and clinical parameters. We then verified transcript content for the control match to ensure study eligibility. First, we reviewed the transcript files of all 820 control candidates using our NLP pipeline. This first verification resulted in the removal of 16 controls, as their transcript files were not machine-readable. Second, we ran the job detection algorithm on the sample. This step was to ensure a proper control condition to the study by confirming the absence of any healthcare worker in the control sample. We hand-labeled transcripts snippets from the remaining 804 potential controls to confirm the absence of medical professionals, using the same labeling procedures as the job algorithm. Results from hand-labeling suggested that 26 candidate controls were potentially HCWs, and were thus removed from the control set. In sum, from the first round of HCWs-controls matches, 42 controls had to be removed during the matching verification phase.

As the matching algorithm produced identifiable 1:1 HCW-control matching pairs (i.e. the *subclass* output), we identified the 42 HCW who didn't have a match anymore following transcript verification. We then reran matching procedures to find an additional 42 non-HCW controls for the remaining 42 unmatched HCWs from the remaining pool of outpatients. First, we removed from the initial sample of candidate outpatients (n=5,980) all previous control matches (n=820), leaving only previously unassigned general population patients (n=5,160). From this pool, we then used the matching algorithm described above to find controls for the 42 unmatched HCWs. This procedure resulted in additional 42 matches that, upon inspection, had analyzable treatment transcripts. Further examination of transcript snippets from the 42 new controls, based on hand-labeling, verified the absence of any medical professionals in the new controls. The final sample consisted of 820 non-HCW matched controls.

Structural Topic Modeling

Transcripts were analyzed using topic modeling, a generative modeling method in Natural Language Processing (NLP) in which semantic themes within a corpus are algorithmically discovered without the need for line-by-line annotation [29]. In brief, topic modeling imagines that every document within a corpus contains a mixture of corpus-wide topics, or, formally defined, distributions of words within a fixed vocabulary. Topic modeling algorithms seek to find the topics that best characterize a given corpus across documents. They thus offer a rapid method for collecting potential themes in a set of texts, as a starting point for more in-depth manual investigation. For collections of texts like transcripts of psychotherapy sessions, they also offer the potential to be more privacy-preserving: while the modeler must typically access the contents of raw transcripts for model development, meaningful analysis can be done on the topics themselves, which are distributions of common words less likely to reveal private information than the raw texts.

We additionally used the structural topic model (STM) in this project, a variant that enables the use of observed per-document covariates in the topic discovery algorithm.[45] This model enables the exploration of how these observed covariates might influence *topic prevalence*, or the proportion of a document associated with a topic, and *topical content*, or the distribution of words used within a topic.

In this proof-of-concept work, we examined *topic prevalence* conditioned on a patient's psychopathology; or, the association between a patient's depression and anxiety symptom levels at intake (the per-document covariate) with the proportion of their messages to therapists devoted to topics around mental health and healthcare work (the topic prevalence). To briefly summarize the STM, the document-generating process envisioned is as follows:

For a document indexed with *d* within a corpus *D,* with a vocabulary of size *V* and an STM with *K* topics:

1. Draw the document-level distribution of topics $\vec{\theta}_d$ from a logistic-normal generalized linear model conditioned on the document-level metadata (X_d):

   $$\vec{\theta}_d \mid X_d\gamma,\ \Sigma \sim LogisticNormal(\mu = X_d\gamma,\ \Sigma)$$

   where $\sum$ is a $(K - 1)$-by-$(K - 1)$ covariance matrix, γ is a p-by-(K - 1) matrix of coefficients, and X_d is a 1-by-p vector.
2. For each word in a document, ($n \in \{1, \ldots, N_d\}$):
   a. Draw the word's topic assignment $z_{d,n}$ from a multinomial distribution conditioned on $\vec{\theta}_d$:

$$z_{d,n} \mid \vec{\theta}_d \sim Multinomial(\vec{\theta}_d)$$

b. Draw a word conditioned on that topic assignment, and the document-specific distribution over words in that topic, $\beta_{d,k=z_{d,n}}$:

$$w_{d,n} \mid z_{d,n}, \beta_{d,k=z_{d,n}} \sim Multinomial(\beta_{d,k=z_{d,n}})$$

Because we did not use the content covariate in our analysis, the document-specific distributions of words representing each topic ($\beta_{d,k=z_{d,n}}$) are point-estimated.

As is in many generative models, the parameters of this process can be estimated from the data using the variational expectation-maximization algorithm. In this work, we conducted estimation using the sped-up approximation of the variational EM housed in the *stm* R package [38]. We conducted all work using Python, version 3.9.9, and R[37], version 4.1.2. For initialization, our models used a spectral decomposition – or non-negative matrix factorization – of the word co-occurrence matrix. We set a maximum iteration of 75.

```
binaryPrevHCW.fit <- stm(prepped.hcw_docs$documents,
     prepped.hcw_docs$vocab,
     K = K, # number of topics
     prevalence =~ `combined_phq_gad_binarized`, # conditioned on anxiety & depression
     max.em.its = 75,
     data = prepped.hcw_docs$meta,
     init.type = "Spectral",
     verbose=FALSE)
```

Topics inferred from the estimation described above can be associated with covariates per the logistic-normal GLM described in (1). For our analysis, we used a first difference estimate, in which topic prevalence for a particular topic is contrasted for two groups within a categorical covariate (none-to-mild vs. moderate-to-severe depression or anxiety). To estimate the parameters of the GLM, we used a global approximation to the average covariance matrix governing the variational posterior (versus a per-document approximation that was less computationally tractable). We also set a ridge penalty of $1x10^{-5}$ on initializing the coefficients for numerical stability (avoiding divide-by-zero errors caused by the sparseness of text data).

```
binaryPrevHCW.effects <- estimateEffect(1:K ~ `combined_phq_gad_binarized`,
                                        binaryPrevHCW.fit,
                                        documents=prepped.hcw_docs$documents,
```

```
                                        meta = prepped.hcw_docs$meta,
                                        uncertainty = "Global",
                                        prior=1e-5)

plot.estimateEffect(binaryPrevHCW.effects,
                   covariate = "combined_phq_gad_binarized",
                   model = binaryPrevHCW.fit,
                   method = "difference",
                   cov.value1 = 1, cov.value2 = 0,
                   xlab = "none to mild <--------> moderate to severe",
                   main="Effect of Anxiety/Depression on Mean Topic Prevalence, HCW set",
                   xlim = c(-.1, .1),
                   labeltype = "frex", # use "frex" to rank top words
                   n = 3, # only list the top 3 words
                   verbose.labels = F, # labels get spammy with this T
                   width = 100 # allow for longer label strings,
                   )
```

The results of the GLM fitting provide association estimates between covariates and topics, which we provide in Supplementary Table 1. Additional R code used for our models is provided in the supplementary computational notebook.

Selecting the number of topics

A key challenge in topic modeling is how a researcher can choose the number of topics *K*. Throughout unsupervised learning, automatic parameter selection towards optimal clustering remains an open research problem, but what is clear is that for semantic research questions like topic or theme generation, automatic selection is by definition insufficient. Grimmer and Stewart [41] argue the goal of a topic model is not to find model-based representations of documents – the paradigm under which maximum likelihood becomes the appropriate evaluation method – but rather to reveal “substantively interesting information” from reductions of those documents achieved via preprocessing. Statistical inference methods for fitting generative models to data tend towards evaluation methods that reflect the generative process; the goal of such techniques is to maximize the likelihood of a dataset held out of the training process for evaluation (e.g., maximizing held-out likelihood or minimizing residuals). But as demonstrated in Chang et al. [40], there can actually often be a *negative* relationship between a model that fits best in terms of held-out likelihood and its ability to provide substantive information to a researcher: words can co-occur because they are similar parts of

speech like prepositions, or for other reasons that do not substantively inform the themes of a text. Thus, statistical techniques for model evaluation must be accompanied by substantive and interpretive human validation, often by having a trained person read representative texts in each topic from a candidate model to interpret the meaning of the clustering.

In this study, we used a mixed statistical and substantive validation process to select *K* for the HCW and non-HCW datasets. For each dataset, we began by running four diagnostic statistical tests for a range of values *K*: (1) held-out likelihood; (2) residuals; (3) semantic coherence; and (4) frequency-weighted exclusivity. As discussed by Chang and colleagues [40], standard evaluation methods in generative modeling, e.g. held-out likelihood and residuals, are likely less useful for producing substantive or semantically meaningful topics. We show them here for completeness, but for decision-making around the ultimate value of *K*, we turned to the evaluation metrics offered in *stm* that the literature shows better align with human judgments of topic quality: semantic coherence and frequency-weighted exclusivity (FREX).

Semantic coherence is a diagnostic test for topic quality that is maximized when the most probable words in a given topic frequently co-occur together. Mimno et al [43] show it correlates with human judgment of topic quality. The frequency-weighted exclusivity (FREX) metric improves on semantic coherence by introducing the notion of a word's exclusivity to a topic, or its usage rate relative to a set of comparison topics. FREX prevents very frequent words (e.g. stopwords) from dominating topic distributions, and is similarly maximized when topic quality is maximized [39].

We ran all four tests for the range of *K*-values from 10-50, hypothesizing that in our data, $K>50$ topics would be too many for meaningful interpretation, and $K<10$ would be too few. For each value *K,* we ran our standard model: a structural topic model fit using spectral initialization with maximum EM iterations set at 75, with topic prevalence within each individual's document set to vary by their binarized psychopathology score.

Supplementary Figure 2 depicts the diagnostic plots for both datasets. As expected, the held-out likelihood curves are noisy for the small and nonstandard documents that are therapy conversations, and the residuals similarly show potential overfitting. Interestingly, semantic coherence shows a steady downward trend in both datasets, perhaps also due to the smaller and less standard nature of the data, while exclusivity increases to a plateau. Based on these plots, we selected for downstream review the sets of topics at $K=20$ and $K=30$ in both the HCW and control models: $K=20$ since semantic coherence seemed high in both models, and $K=30$ since it seemed for both models to be at a reasonable plateau in exclusivity. To select the final *K* for each, we then used manual inspection of the topics resulting from each model, discussed below.

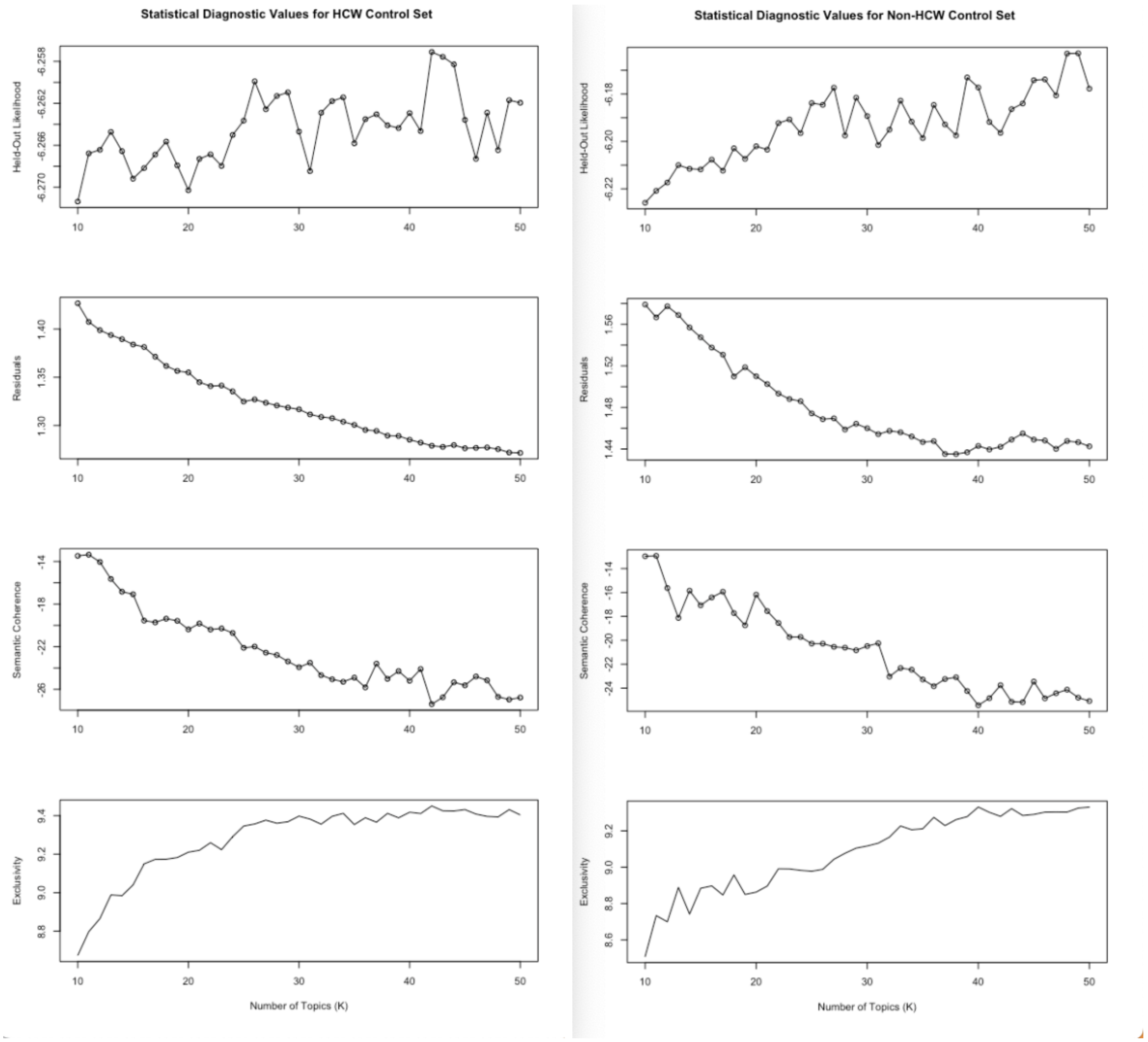

Supplementary Fig. 2. Statistical diagnostic tests used to select *K*, the number of topics, for the HCW and control transcripts corpora.

Characterizing relevant topics.

Topics are defined as distributions over terms; however, there exist a number of ways to characterize individual topics and sets of topics once they have been inferred. In this work, we chose to characterize topics via manual inspection of the top 10 most characteristic terms within them, according to their frequency-weighted exclusivity score (FREX). As discussed in Airoldi & Bischof,[39] this is the harmonic mean of a term's rank by within-topic probability (frequency) and its rank by distribution of topic given word (exclusivity).

$$\mathrm{FREX}_{k,v} = \left(\frac{\omega}{\mathrm{ECDF}(\beta_{k,v} / \sum_{j=1}^{K} \beta_{j,v})} + \frac{1-\omega}{\mathrm{ECDF}(\beta_{k,v})} \right)^{-1}$$

Following the defaults in the stm package, we set the weight *w* to 0.7 to slightly favor exclusivity. The resulting FREX-ranked lists of terms were then used as a starting point for understanding the content of a given topic and set of topics. One researcher (ET) read through the list of FREX-ranked words for each candidate model to determine a suitable set of distributions. ET also consulted the top documents within the corpus associated with a given topic to better understand candidate topics.

As is standard in topic modeling approaches to text analysis, some topics were easily interpretable as content relevant to therapy; e.g., topic 2 in the HCW dataset clearly indicates discussion of panic attacks and anxiety (Figure 1). Others clearly demonstrate terms grouped together for functional reasons; e.g., topic 23 in the final HCW dataset ('video, today, session') and topic 8 in the final control dataset ('thank, session, video') indicate patients scheduling sessions with their therapists. Some topics did require closer inspection, as they seemed to contain overlap between semantically distinct topics. As an example, topic 30 in the final HCW dataset seemed to contain discussion of death and grief, and also credit card processing (Table 1).

From review of the sets of topics at *K=20* and *K=30,* **we selected *K=30* as the model for analysis in both HCW and control datasets**. In line with the literature on topic quality [40], we determined both sets adequately captured relevant topics, and *K=30* appeared from manual inspection to achieve better separability, or distinction between concepts, within topics – that is, there were fewer topics that contained distinct concepts.

Having decided on the model to take forward in analysis (*K=30* for both sets), we then used FREX-ranked lists of terms to cull the list of topics to those most relevant to the research questions. Relevance was determined based on absolute agreement between one researcher (ET), two doctoral-level clinical psychologists (MM and TDH), and one psychiatrist (NMS) based on FREX-ranked words. For example, in the HCW dataset, topic 4, in which the top 10 terms by FREX are 'unit, hospit, icu, nurs, virus,

news, sick, covid, safe, fear', was deemed relevant to the research questions, but topic 23, in which the top 10 words by FREX are 'video, today, session, tomorrow, morn, ill, yesterday, weekend, plan, chat', was deemed less relevant for the purpose of this analysis.

The topics deemed relevant for our research question are reported in the main manuscript (Table 2). In the Supplementary here, we describe all topics for completeness. Supplementary Tables 1 and 2 depict the top 10 words by FREX within each topic for the HCW and control datasets respectively. Supplementary Figures 3 and 4 depicts the expected proportions for all topics in both datasets.

Supplementary Results from Topic Modeling

Supplementary Table 1. Full set of topics generated by the HCW model, described in terms of the top 10 terms in each topic by FREX score.

Topic	Top 10 terms by FREX score	Relevant to mental health?	Relevant to healthcare / pandemic?
H1	*explain, see, eye, appreci, middl, insid, separ, affect, process, someon*		
H2	*panic, breath, attack, symptom, anxious, anxieti, exercis, chest, tool, calm*	Yes	
H3	*covid, worker, healthcar, hospit, patient, physician, current, week, promot, doctor*		Yes
H4	*unit, hospit, icu, nurs, virus, news, sick, covid, safe, fear*		Yes
H5	*schedul, boss, refer, set, staff, manag, allow, depart, hour, honest*		Yes
H6	*therapist, therapi, provid, npi, free, health, recent, servic, prefer, due*		
H7	*wife, nurs, travel, year, assign, ago, click, regist, goal, practition*		
H8	*thank, sorri, match, messag, let, hello, check, pleas, repli, hope*		
H9	*great, love, yes, perfect, help, happi, wors, can, anxieti, late*		
H10	*eat, dog, food, drink, usual, yoga, walk, hike, weight, activ*		
H11	*lol, stuff, yeah, super, kinda, guess, honest, idk, got, know*		
H12	*okay, option, yeah, cri, wait, hey, sound, licens, adjust, number*		
H13	*sleep, night, bed, shift, asleep, wake, usual, fall, morn, relax*	Yes	
H14	*boyfriend, famili, space, member, distanc, alon, spend, friend, weekend, enjoy*		
H15	*depress, feel, mood, anyth, suicid, quarantin, sad, episod, sometim, hard*	Yes	
H16	*patient, mask, test, shift, unit, wear, staff, icu, ppe, coronavirus*		Yes
H17	*relationship, hurt, sex, cheat, pain, togeth, trust, happi, boyfriend, love*		
H18	*husband, son, kid, babi, daughter, pregnant, hous, mother, children, law*		
H19	*abus, divorc, marri, privat, confid, self, marriag, esteem, date, troubl*		
H20	*someth, emot, other, feel, wrong, think, sometim, sens, thought, peopl*		
H21	*stress, challeng, increas, relief, level, team, stressor, overal, focus, line*	Yes	
H22	*guess, pretti, relationship, sort, partner, differ, though, realiz, probabl, term*		
H23	*video, today, session, tomorrow, morn, ill, yesterday, weekend, plan, chat*		
H24	*marriag, argument, convers, perspect, communic, fight, anger, kid, behavior, angri*		
H25	*mom, sister, brother, dad, parent, reflect, grow, alcohol, famili, drink*		
H26	*return, went, play, hous, came, tell, clean, girl, birthday, parti*		
H27	*job, move, wed, money, pay, plan, fiancã, career, chose, new*		
H28	*said, told, ask, talk, felt, text, embarrass, date, letter, want*		
H29	*resid, remain, attend, program, becom, answer, clinic, mayb, mean, studi*		Yes
H30	*death, card, die, grief, code, credit, pass, deal, charg, enter*	Yes	

Supplementary Table 2. Full set of topics generated by the control model, described in terms of the top 10 terms in each topic by FREX score.

Topic	Top 10 terms by FREX score	Relevant to mental health?	Relevant to healthcare / pandemic?
C1	relationship, boyfriend, togeth, partner, date, selfish, girlfriend, futur, colleg, spend		
C2	anger, forgiv, discuss, hurt, angri, behavior, intak, lie, said, sexual	Yes	
C3	guy, text, repli, date, messag, block, reach, stop, attract, men		
C4	hello, live, lower, card, curious, link, abl, see, offer, purchas		
C5	tire, leav, offic, sleep, busi, teach, super, want, guilt, asleep		
C6	readi, night, today, said, cri, yesterday, went, dog, hous, sad		
C7	complet, list, pull, point, note, failur, mistak, mention, exampl, part		
C8	session, tomorrow, avail, thank, chat, doubt, video, messag, schedul, free		
C9	therapi, depress, therapist, issu, anxieti, disord, eat, month, cost, coupl	Yes	
C10	yeah, school, stuff, class, kinda, pretti, guess, bad, colleg, gon		
C11	nice, quarantin, late, gym, enjoy, crazi, glad, weather, excit, heavi		Yes
C12	year, name, ago, water, wall, great, near, order, smoke, regular		
C13	meet, place, due, regard, speak, contact, addict, virus, avoid, present		
C14	husband, old, marri, divorc, mother, babi, marriag, pregnant, son, father		
C15	okay, yes, age, call, phone, talk, ill, pleas, sorri, someon		
C16	felt, feel, self, negat, anxious, thought, sad, bad, scare, boyfriend	Yes	
C17	kind, sort, sometim, guess, game, though, actual, pretti, mayb, usual		
C18	said, say, control, ask, emot, trust, wrong, someth, believ, told		
C19	die, experienc, current, attack, medic, alcohol, rate, daili, health, panic	Yes	
C20	often, communic, moment, exampl, express, convers, sens, tend, someth, confid		
C21	breath, sleep, panic, sick, attack, night, anxious, anxieti, worri, calm	Yes	
C22	kid, result, daughter, son, babi, hous, play, bed, clean, children		
C23	pay, job, money, career, bill, paid, financi, busi, appli, compani		
C24	great, good, sound, wife, morn, pretti, young, forward, well, bit		
C25	pandem, concern, anxieti, situat, cope, corona, group, relat, social, extrem		Yes
C26	dad, mom, pass, brother, sister, hous, parent, abus, grow, alway		
C27	team, manag, boss, project, task, routin, offic, work, cowork, hour		Yes
C28	earlier, peopl, boy, girl, everyon, side, stand, believ, man, women		
C29	comfort, drink, definit, weight, social, date, super, caus, hang, friend		
C30	decis, process, want, accept, best, figur, space, solut, choic, decid		

Supplementary Fig. 3. Expected topic proportions in Healthcare Workers (HCW) and general population matched control samples.

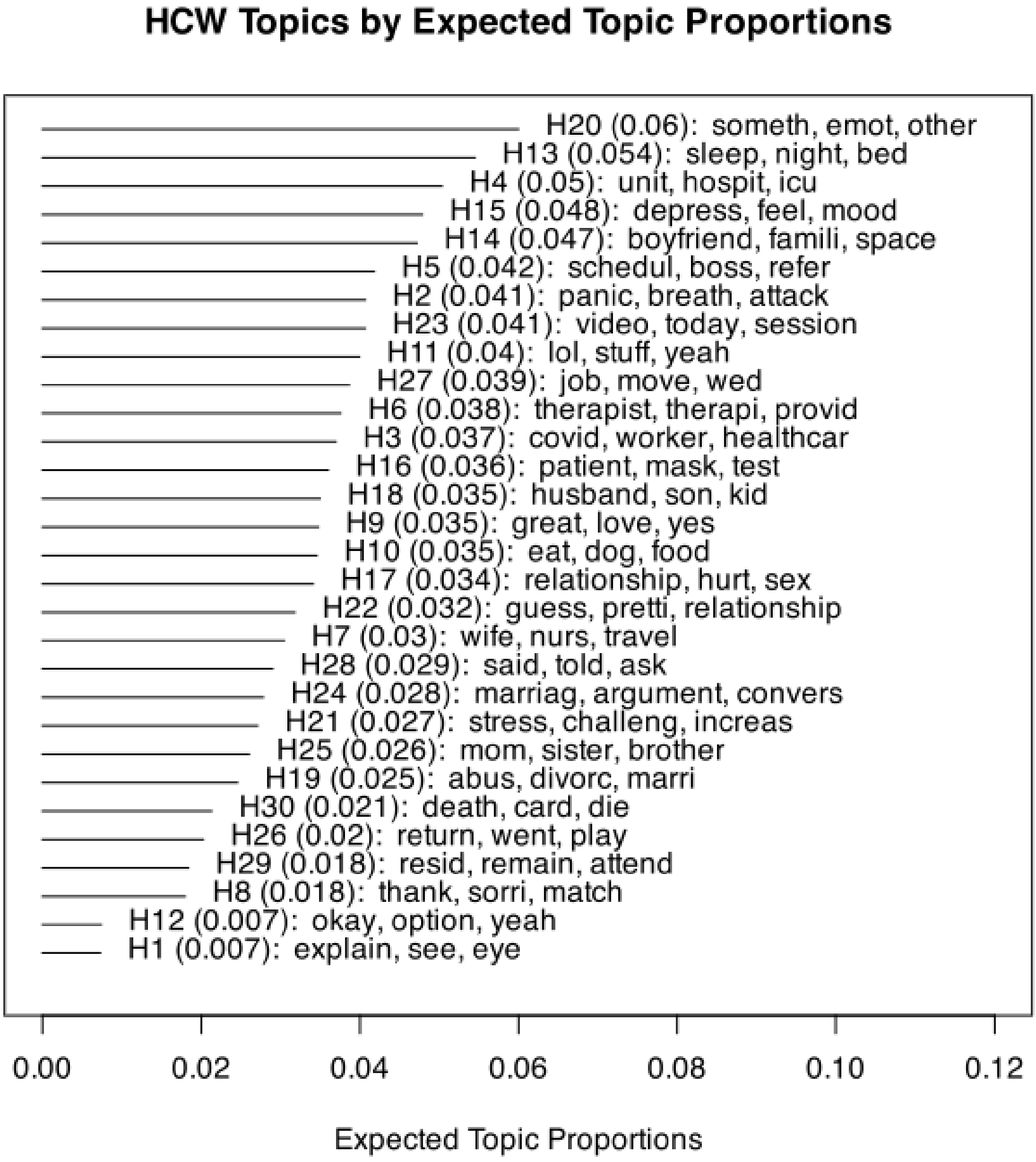

Control Topics by Expected Topic Proportions

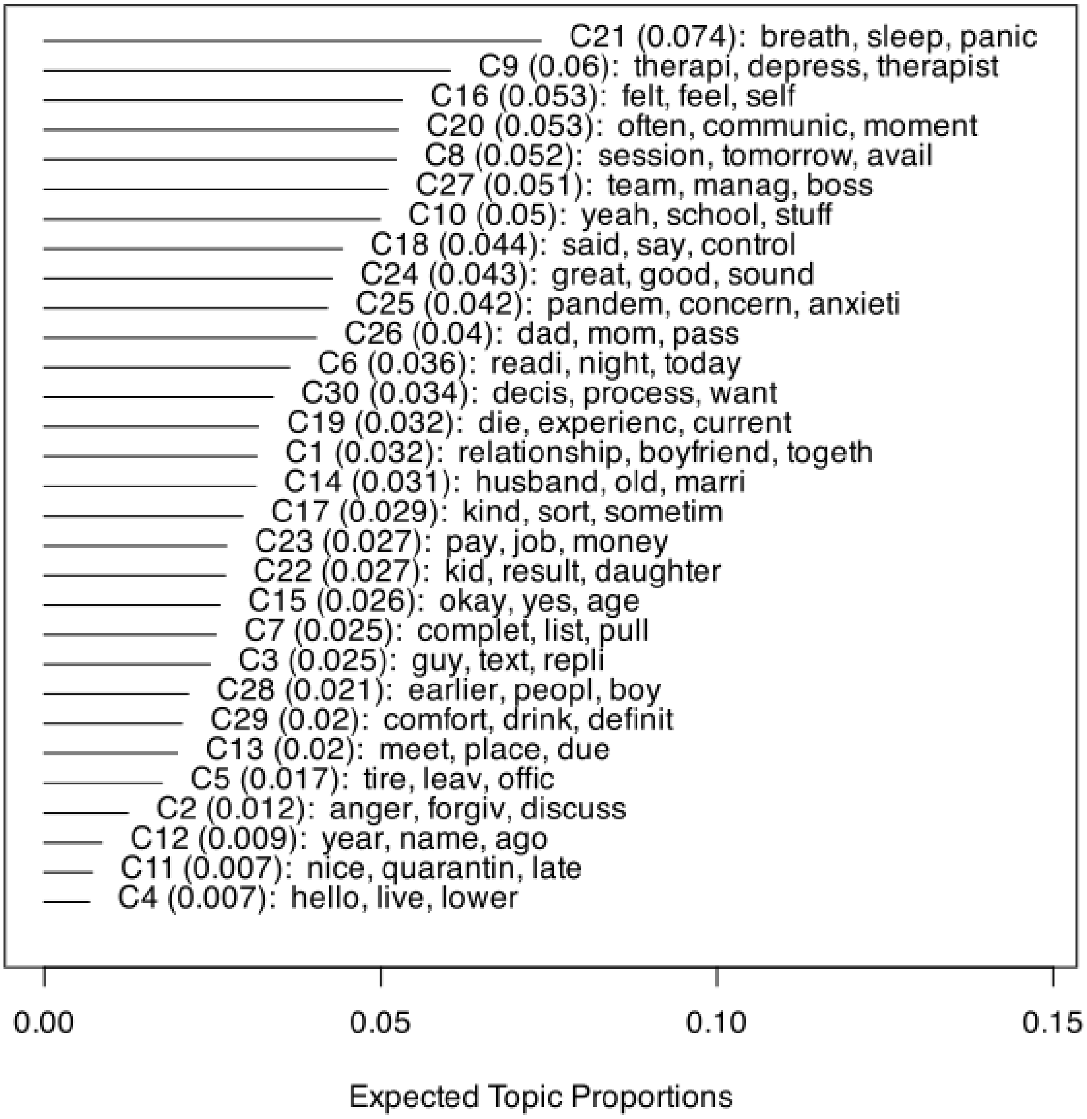

Supplementary Fig. 4. Estimates of STM association with moderate to severe anxiety and/or depression for all 30 topics.

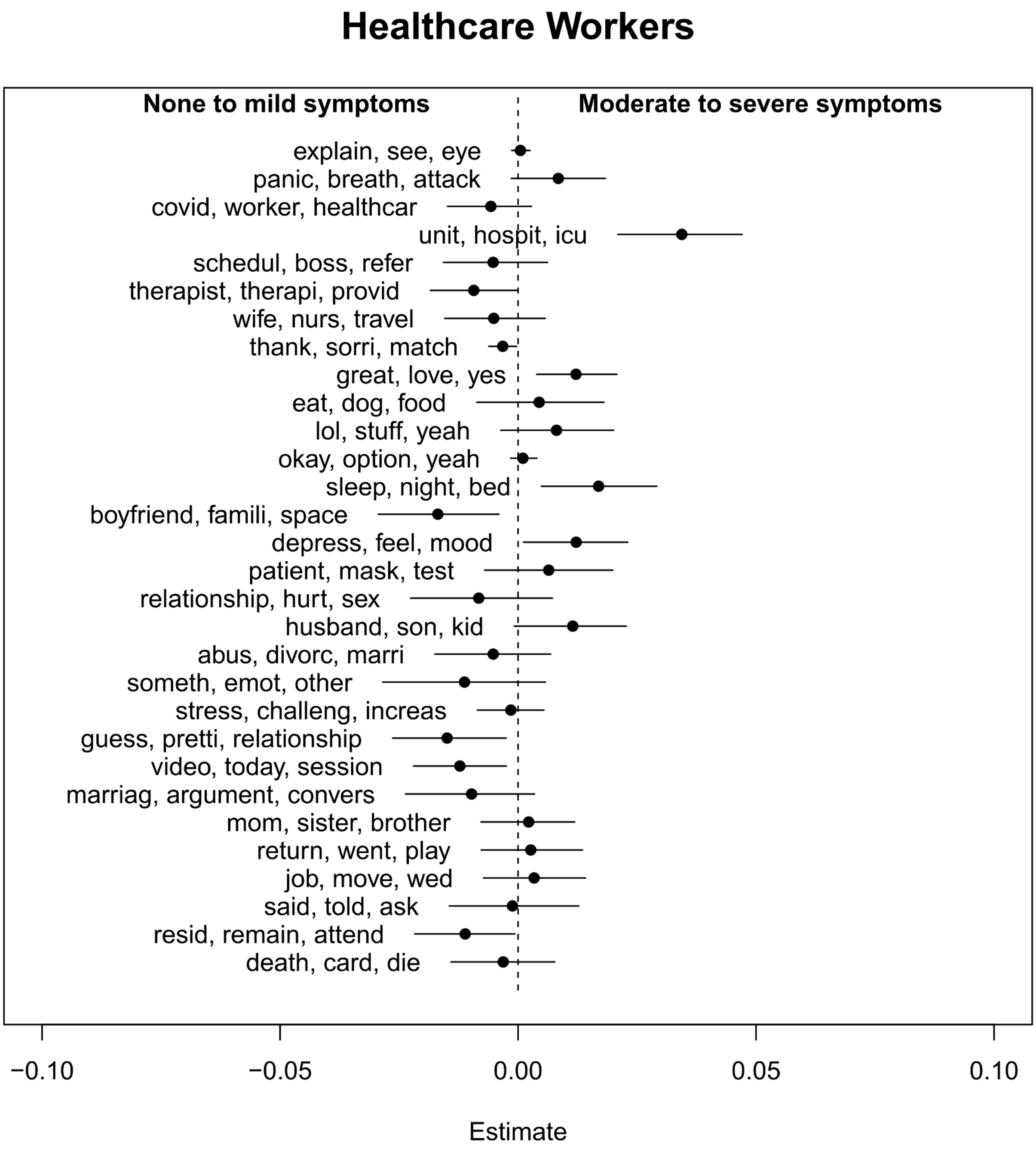

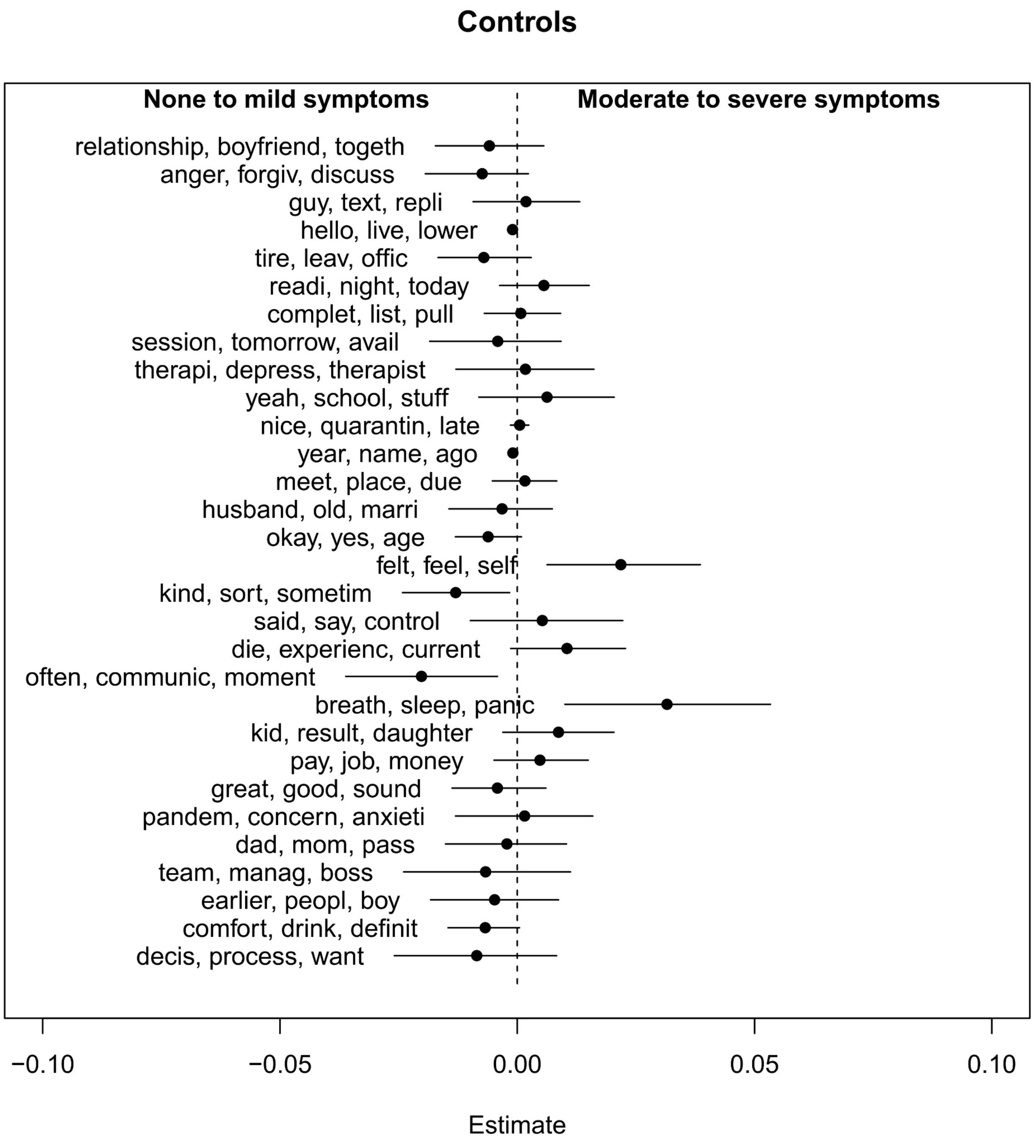

Controls
None to mild symptoms
Moderate to severe symptoms
relationship, boyfriend, togeth
anger, forgiv, discuss
guy, text, repli
hello, live, lower
tire, leav, offic
readi, night, today
complet, list, pull
session, tomorrow, avail
therapi, depress, therapist
yeah, school, stuff
nice, quarantin, late
year, name, ago
meet, place, due
husband, old, marri
okay, yes, age
felt, feel, self
kind, sort, sometim
said, say, control
die, experienc, current
often, communic, moment
breath, sleep, panic
kid, result, daughter
pay, job, money
great, good, sound
pandem, concern, anxieti
dad, mom, pass
team, manag, boss
earlier, peopl, boy
comfort, drink, definit
decis, process, want
−0.10
−0.05
0.00
0.05
0.10
Estimate

Supplementary Table 3. Numerical estimates for topic association with moderate to severe anxiety and/or depression.

Topic	Healthcare Workers (n=820)			
	Terms	EST	95% CI	P
H1	*explain, see, eye*	.001	±.002	.594
H2	*panic, breath, attack*	.009	±.01	.107
H3	*covid, worker, healthcar*	-.007	±.009	.140
H4	*unit, hospit, icu*	.035	±.013	<.001
H5	*schedul, boss, refer*	-.005	±.01	.369
H6	*therapist, therapi, provid*	-.009	±.009	.049
H7	*wife, nurs, travel*	-.004	±.01	.398
H8	*thank, sorri, match*	-.003	±.003	.038
H9	*great, love, yes*	.012	±.009	.009
H10	*eat, dog, food*	.003	±.015	.664
H11	*lol, stuff, yeah*	.009	±.012	.153
H12	*okay, option, yeah*	.001	±.003	.538
H13	*sleep, night, bed*	.016	±.014	.024
H14	*boyfriend, famili, space*	-.019	±.013	.006
H15	*depress, feel, mood*	.014	±.012	.028
H16	*patient, mask, test*	.006	±.014	.400
H17	*relationship, hurt, sex*	-.01	±.015	.174
H18	*husband, son, kid*	.012	±.011	.043
H19	*abus, divorc, marri*	-.005	±.013	.403
H20	*someth, emot, other*	-.012	±.016	.152
H21	*stress, challeng, increas*	-.001	±.007	.721
H22	*guess, pretti, relationship*	-.015	±.013	.022
H23	*video, today, session*	-.011	±.01	.025
H24	*marriag, argument, convers*	-.01	±.013	.130
H25	*mom, sister, brother*	.003	±.01	.552
H26	*return, went, play*	.002	±.01	.643
H27	*job, move, wed*	.005	±.011	.371
H28	*said, told, ask*	0	±.014	.993
H29	*resid, remain, attend*	-.011	±.011	.052
H30	*death, card, die*	-.003	±.01	.511

Topic	Matched controls (n=820)			
	**Terms**	**EST**	**95% CI**	***P***
C1	*relationship, boyfriend, togeth*	-.005	±.012	.445
C2	*anger, forgiv, discuss*	-.006	±.01	.193
C3	*guy, text, repli*	.001	±.011	.839
C4	*hello, live, lower*	-.001	±.001	.04
C5	*tire, leav, offic*	-.007	±.009	.172
C6	*readi, night, today*	.006	±.009	.221
C7	*complet, list, pull*	.002	±.008	.704
C8	*session, tomorrow, avail*	-.004	±.014	.59
C9	*therapi, depress, therapist*	0	±.015	.957
C10	*yeah, school, stuff*	.004	±.016	.581
C11	*nice, quarantin, late*	0	±.002	.707
C12	*year, name, ago*	-.001	±.001	.095
C13	*meet, place, due*	.001	±.007	.747
C14	*husband, old, marri*	-.003	±.011	.644
C15	*okay, yes, age*	-.006	±.007	.087
C16	*felt, feel, self*	.021	±.016	.009
C17	*kind, sort, sometim*	-.013	±.012	.045
C18	*said, say, control*	.004	±.015	.595
C19	*die, experienc, current*	.011	±.012	.077
C20	*often, communic, moment*	-.021	±.017	.017
C21	*breath, sleep, panic*	.031	±.022	.007
C22	*kid, result, daughter*	.007	±.01	.198
C23	*pay, job, money*	.004	±.01	.421
C24	*great, good, sound*	-.005	±.011	.391
C25	*pandem, concern, anxieti*	.003	±.015	.673
C26	*dad, mom, pass*	-.002	±.013	.772
C27	*team, manag, boss*	-.005	±.016	.546
C28	*earlier, peopl, boy*	-.003	±.011	.623
C29	*comfort, drink, definit*	-.007	±.008	.095
C30	*decis, process, want*	-.009	±.015	.22

Note. Terms = Top 3 FREX terms per topic; EST = Regression coefficient estimates; 95% CI = 95% Confidence Interval.

Healthcare profession detection algorithm

HCWs included in the study were referred to the digital health platform as part of a promotion offering one month of free treatment [48]. HCW were defined as healthcare and medical providers (e.g., physicians, nurses, residents, EMS, social workers, etc.) with an active National Provider Identifier (NPI) profile at the time of treatment. Eligibility was verified by the platform through employment and NPI verification.

Information on the occupations of the HCWs in the sample was needed for the study to determine the distribution of medical professions, as well as to identify the portion of patient-facing HCW professions (e.g., how many physicians and nurses vs. admins). However, for privacy reasons, individual employment information and NPI numbers were not available as data for our study, since NPI are unique person-specific identifiers. Given the high likelihood of workplace-related distress endorsed during treatment in the wake of the COVID-19 pandemic,[5] we used treatment transcripts as the information source to infer medical professions in the HCW sample. Information extraction used NLP methods, given prior success of named entity recognition (NER) applications to identify occupational roles from unstructured text [42, 44, 46]. Moreover, NER was chosen thanks to the ability to extract occupational information while minimizing access to the de-identified treatment transcripts, thus further preserving patient privacy. Algorithm characteristics and applications are further described below.

Job detection heuristics

We developed a NER heuristic algorithm to detect instances in the transcripts where patients identified as healthcare workers or spoke about their professional roles. The NER algorithm used simple string matching procedures to map grammatical cues to their following job nouns. Grammatical cues included, for example, "I am a" , "I work as" , "my work as" , "my job as", "being a" , "I practice". Job nouns consisted of healthcare professions categorized based on keywords derived from healthcare roles, their abbreviations, and medical specialties (e.g., nurse = nurse, nursing, NP, RN, CNA, LVN, APRN, LPN, ACNP, CNS, ANP, FNP, GNP, PNP, TCRN). Job nouns were listed both with and without capitalizations. Code for the algorithm, grammatical cues, and categorized job nouns token are further reported in the NLP analytic code.

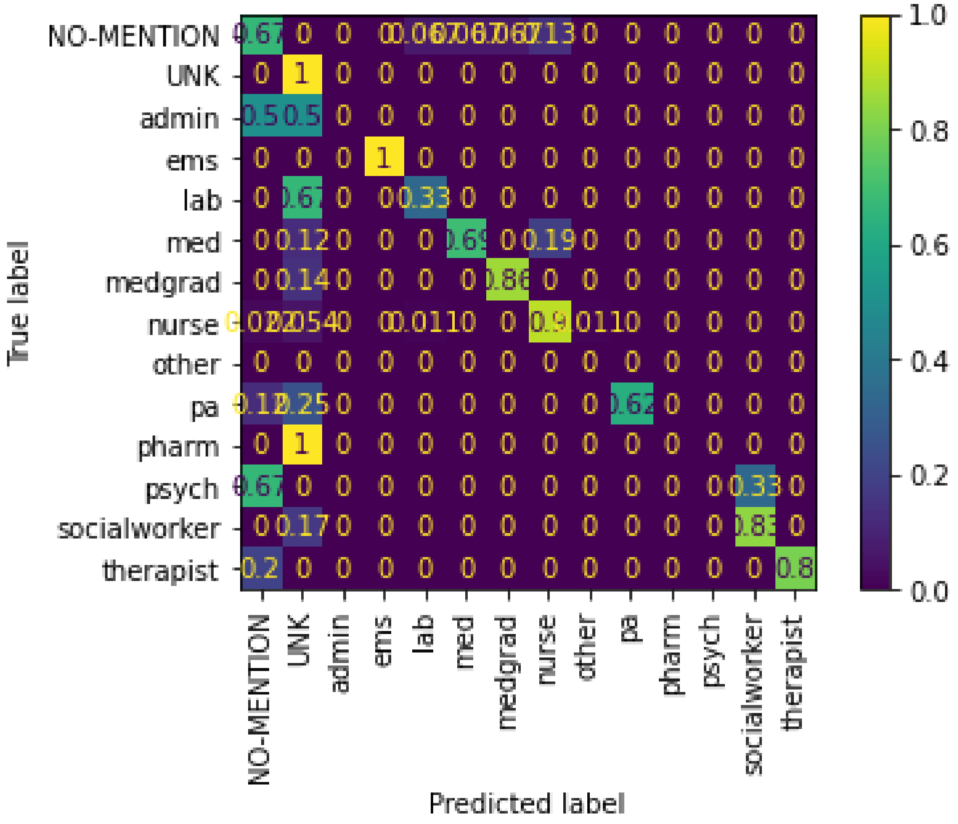

Supplementary Fig. 5. Confusion matrix for the job role identification heuristic algorithm, run on a random sample of n=162 HCWs hand-labeled by the research team.

We verified functionality of the algorithm by comparing its results with hand-labeled findings as ground truth in a random sample of 162 HCW transcripts. Hand-labeling focused on text snippets consisting of utterances containing grammatical cues identified by the algorithm, or a portion of transcripts in the event that the algorithm didn't identify any. Transcripts were annotated by two clinical researchers (MM and ET), respectively with training in psychology and in linguistics.

Using the manual labels as ground truth, we assessed the performance of the algorithm in two parts: (1) accuracy with respect to determining the presence or absence of an HCW job role (e.g., "does the algorithm identify whether a patient mentions a healthcare profession at all?"), and (2) accuracy with respect to determining precisely which HCW profession was mentioned, if any (e.g., "does the algorithm correctly identify when a patient is a nurse vs a doctor, etc?"). Comparison between automated and manual labels indicated that 134/162 (82.72%) of the sample was correctly identified to contain a mention of a HCW job role, or make no mention of the patient's job at all (as not all HCWs stated their professions in the course of therapy). Of the 123 patients who stated a HCW job role, the algorithm correctly

identified the specific profession in 117 cases (95.12%). Combining these two accuracies into one metric, the algorithm correctly identifies a specific HCW profession or the absence of a mention of an HCW job in 128/162 cases (79.01%).

Supplementary Table 4. Performance of HCW profession identification algorithm assessed on a hand-labeled sample of HCWs (n=162).

	**Hand-labeled ground truth**	
**Algorithm prediction**	Mentions HCW job role (n=146)	No mention of HCW job role (n=16)
Mentions HCW job role	Correct specific profession: 117<br>Incorrect specific profession: 6	5
No mention of HCW job role	23	11

Job detection in HCW sample

We used the job detection algorithm on the broader set of 820 HCWs as a starting point for identifying the breakdown of professions within our study population. The heuristic algorithm's initial predictions for the are depicted in Supplementary Figure 5. Based on the algorithm results, nearly half the HCWs in our study were in nursing roles (including nurse practitioners, nursing assistants, and registered nurses (see code for a full list of roles). Approximately 16.5% were physicians or resident physicians (including specialists), which we aggregated in one category.

For samples in which the algorithm initially could not identify a job mention (NO-MENTION, n=89), or could not disambiguate between equally likely options (UNK, n=71), we hand-labeled job profession to ensure accuracy. We added the hand-labeled information to the profession count. The resulting final counts for HCW professions are reported in the manuscript (Fig. 2 in main document).

Supplementary Fig. 6. Initial count of HCW professions within the study population, as identified by the heuristic algorithm (n=820)**.**

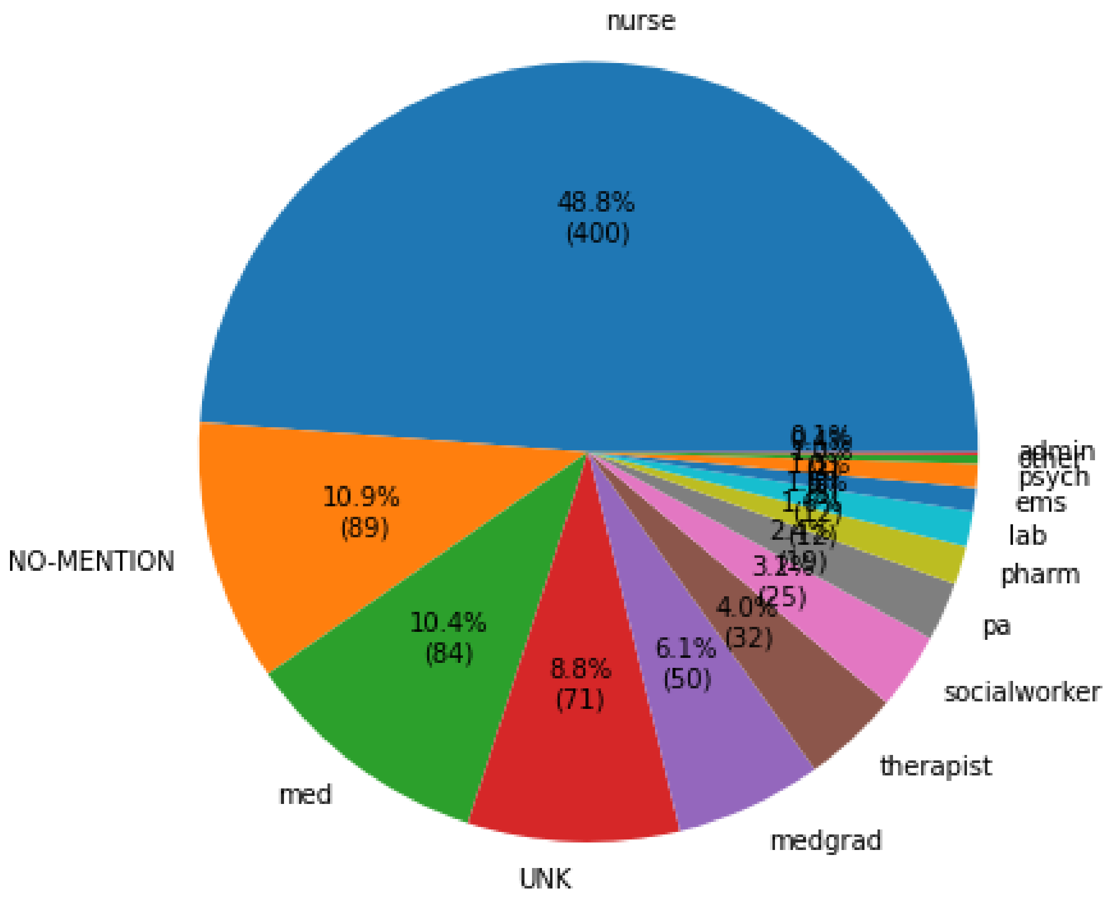

Supplement: Multimedia Appendix 1 [file ai_v2i1e47223_app1.pdf]
