# Supplementary material for: Association of Health Care Work With Anxiety and Depression During the COVID-19 Pandemic: Structural Topic Modeling Study
Source: JMIR AI. 2023 Oct 24;2:e47223. doi: 10.2196/47223 (PMC11041488; doi:10.2196/47223)

Characterizing Topics in Online Psychotherapy During COVID-19 Pandemic

Code

- 1 Load the necessary libraries
- 2 Read in and preprocess the data
- 3 Prepare the outcomes
- 4 Fit the models
  - 4.1 Select K
  - 4.2 Run the models
  - 4.3 Alternatively, start from a loaded model here
- 5 Explore the result
  - 5.1 Plot topics by prevalence
  - 5.2 Examine topic labels
  - 5.3 Examine source documents
  - 5.4 Compare topic vocabularies
- 6 Investigate covariate/topic relationships
  - 6.1 Fit effects
  - 6.2 Plot the estimated topic prevalence differences.

1 Load the necessary libraries

Hide

```
library(stm)
library(data.table)
library(readr)
library(tm)
library(wordcloud)
library(igraph)
```

2 Read in and preprocess the data

This notebook is set up to work with separate data files for the HCW and controls (non-HCW)cohorts. In both cases, the data should be set up with the following fields.

Field	Type	Description
toks	chr	tokenized text of the document, used in modeling
txt	chr	text of the document, provided for debugging
phq	dbl	PHQ-9 scores
gad	dbl	GAD-7 scores

Hide

```
hcw_docs <- read_csv("data/hcw-txts-patient-weeks3-phqsonly.csv")
nonhcw_docs <- read_csv("data/HCWcontrols_2022-09-05.csv")
```

We first load the transcript files and then produce a *textProcessor* object for each of the cohorts. The **textProcessor()** function removes stopwords in English, stems words, removes tokens under 3 characters, and lowercases all tokens. (We should do this preprocessing already as part of tokenization, added here just in case.) We additionally add a few more specific stopwords to the dictionary.

Hide

```
new.stopwords <- c(c("name", "like", "just", "really"), quanteda::stopwords("english"))
processed.hcw_docs <- textProcessor(hcw_docs[["toks"]], metadata = hcw_docs, customstopwords = new.stopwords, stem=TRUE)
```

```
Building corpus...
Converting to Lower Case...
Removing punctuation...
Removing stopwords...
Remove Custom Stopwords...
Removing numbers...
Stemming...
Creating Output...
```

Hide

```
processed.nonhcw_docs <- textProcessor(nonhcw_docs[["toks"]], metadata = nonhcw_docs, customstopwords = new.stopwords, stem=
TRUE)
```

```
Building corpus...
Converting to Lower Case...
Removing punctuation...
Removing stopwords...
Remove Custom Stopwords...
Removing numbers...
Stemming...
Creating Output...
```

As we saw above, we want to remove the rarer words from our corpus so that the algorithm can focus on words occuring in many different documents. The **prepDocuments()** function will do that for us, but to get there, we need to set thresholds on rare words. The **plotRemoved()** function will let us preview how many documents, words, and tokens we'll remove by setting the threshold number of documents to various levels.

Hide

```
# this looks at the first 500 documents
plotRemoved(processed.hcw_docs$documents, lower.thresh = seq(1, 500, by = 5))
```

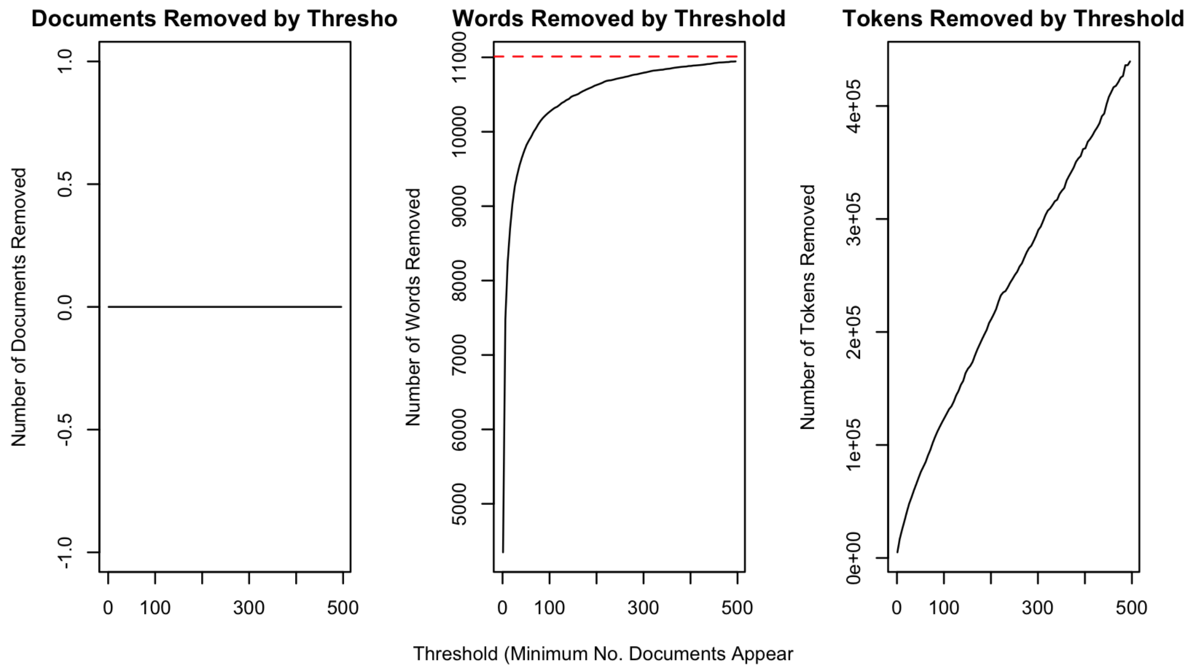

Hide

```
plotRemoved(processed.nonhcw_docs$documents, lower.thresh = seq(1, 500, by = 5))
```

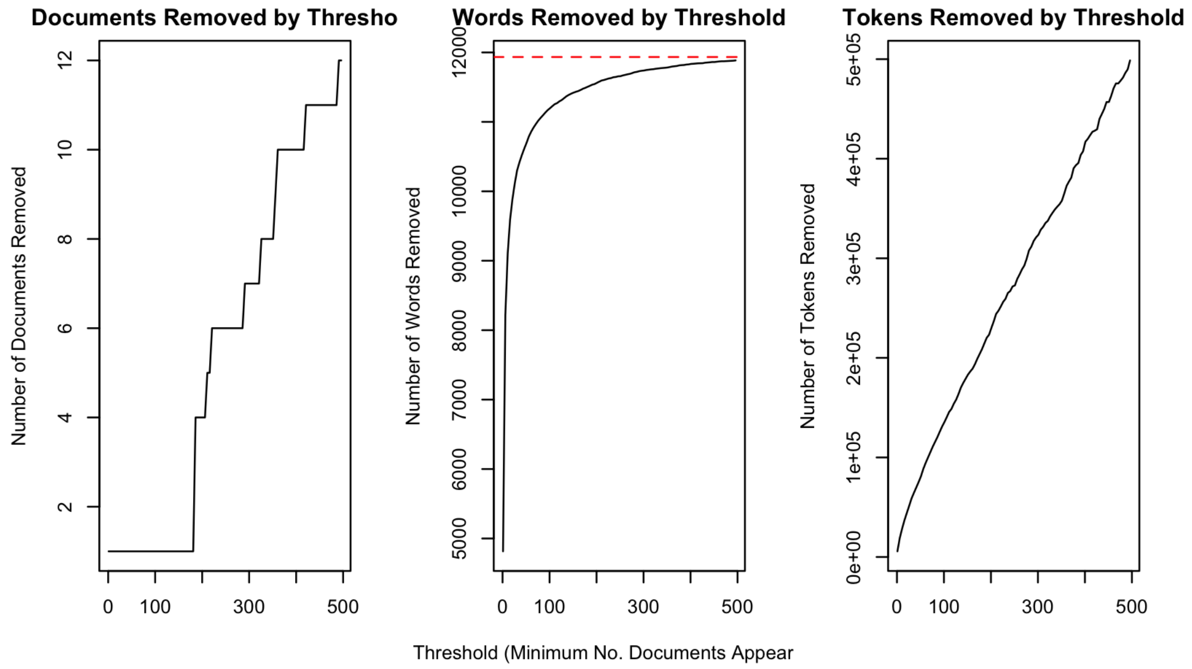

prepDocuments() will handle re-indexing the vocabulary and the metadata as words are removed.

Hide

```
prepped.hcw_docs <- prepDocuments(processed.hcw_docs$documents,
                     processed.hcw_docs$vocab,
                     processed.hcw_docs$meta,
                     lower.thresh = 50
                     )
```

```
Removing 9803 of 11011 terms (59163 of 284382 tokens) due to frequency
Your corpus now has 820 documents, 1208 terms and 225219 tokens.
```

Hide

```
prepped.nonhcw_docs <- prepDocuments(processed.nonhcw_docs$documents,
                     processed.nonhcw_docs$vocab,
                     processed.nonhcw_docs$meta,
                     lower.thresh = 50
                     )
```

```
Removing 10675 of 11934 terms (61450 of 278771 tokens) due to frequency
Removing 1 Documents with No Words
Your corpus now has 817 documents, 1259 terms and 217321 tokens.
```

We've now prepped our documents. We'll save some descriptive data on the document distributions for later.

3 Prepare the outcomes

Next we'll prepare our binarized combination of PHQ-9 and GAD-7 scores outcome variable. PHQ-9 scores ≥ 10 have good sensitivity and specificity for moderate to severe Depression. GAD-7 scores ≥ 10 have good sensitivity for moderate to severe Anxiety. We used these cutoffs to identify the presence of clinically significant psychopathology.

Hide

```
prepare_combined_outcome <- function(docs) {
  meta <- docs$meta

  # Retrieve and binarize the PHQ values
  phq_binarized = c()
  for (phq in meta$phq) {
    if (phq >= 10) {
      phq_binarized <- c(phq_binarized, 1)
    } else {
      phq_binarized <- c(phq_binarized, 0)
    }
  }

  # Retrieve and binarize the GAD values
  gad_binarized = c()
  for (gad in meta$gad) {
    if (gad >= 10) {
      gad_binarized <- c(gad_binarized, 1)
    } else {
      gad_binarized <- c(gad_binarized, 0)
    }
  }

  # Combine PHQ and GAD
  combined_phq_gad_binarized = c()
  for (i in 1:length(meta$gad)) {
    if (phq_binarized[[i]] == 1 || gad_binarized[[i]] == 1) {
      combined_phq_gad_binarized <- c(combined_phq_gad_binarized, 1)
    } else {
      combined_phq_gad_binarized <- c(combined_phq_gad_binarized, 0)
    }
  }

  # Add to meta
  meta$phq_binarized = phq_binarized
  meta$gad_binarized = gad_binarized
  meta$combined_phq_gad_binarized = combined_phq_gad_binarized
  docs$meta <- meta

  return(docs)
}

prepped.hcw_docs <- prepare_combined_outcome(prepped.hcw_docs)
prepped.nonhcw_docs <- prepare_combined_outcome(prepped.nonhcw_docs)
```

Then plot the distributions of our binarized outcomes.

Hide

```
print_distributions <- function(docs, plot_title) {
  par(mfrow=c(1,3))
  hist(docs$meta$phq_binarized, col="lightblue", main="Binarized PHQ")
  hist(docs$meta$gad_binarized, col="lightgreen", main="Binarized GAD")
  hist(docs$meta$combined_phq_gad_binarized, col="purple", main="Binarized psychopathology")
}

print_distributions(prepped.hcw_docs)
```

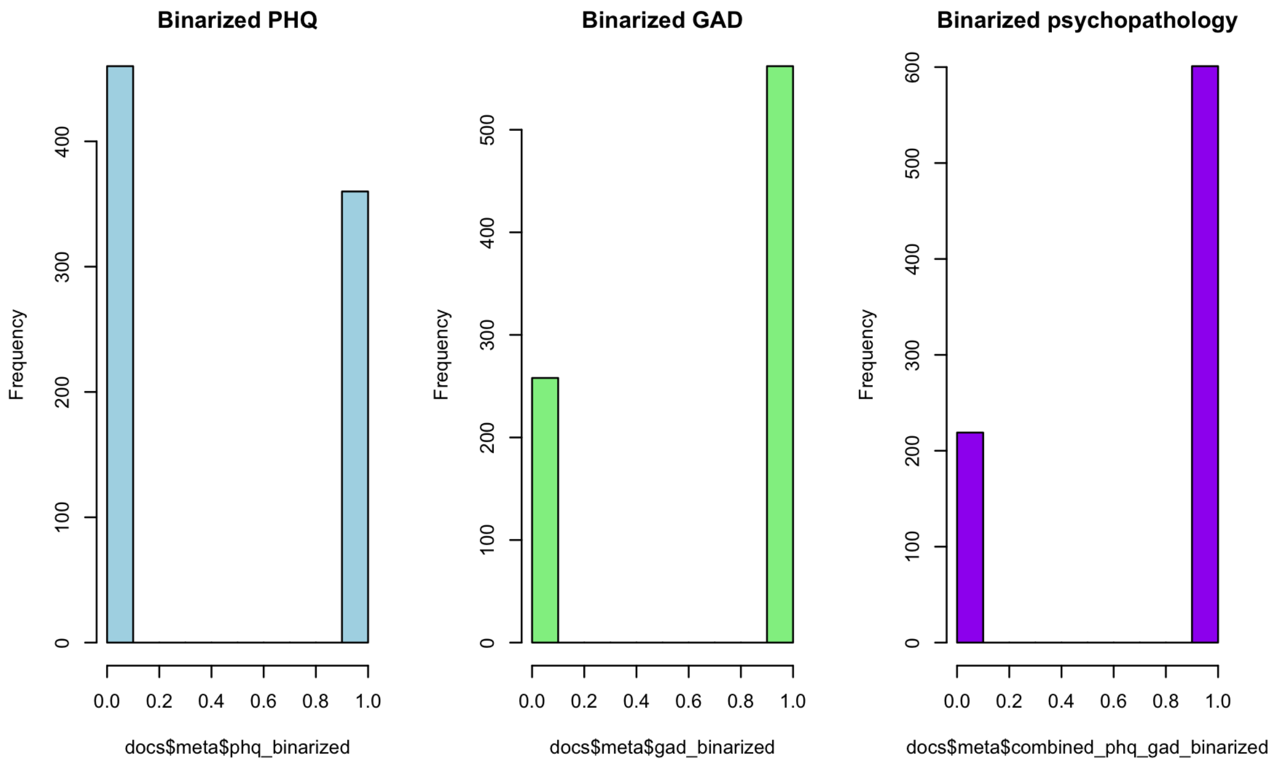

Hide

```
print_distributions(prepped.nonhcw_docs)
```

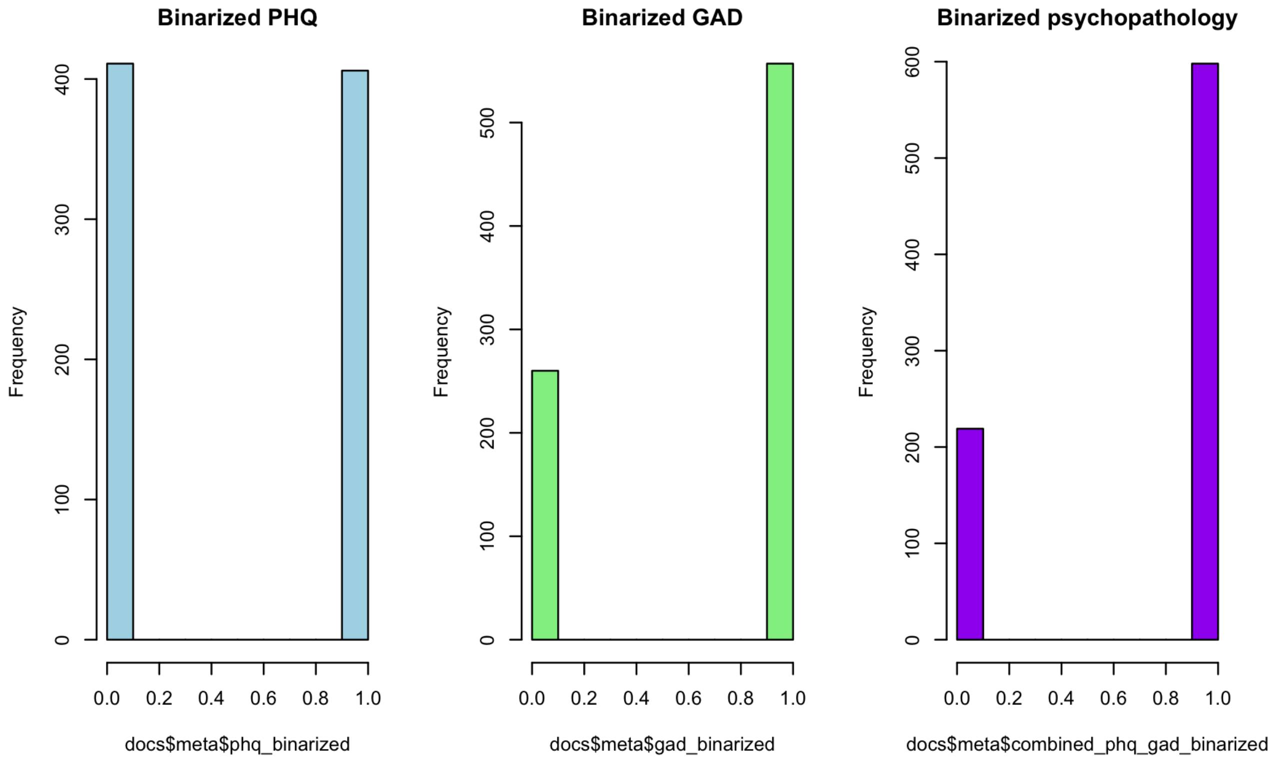

Having prepared our data, we can now model it.

4 Fit the models

The **stm()** function fits a topic model to our data. A few of the parameters to highlight:

1. The overall *topic prevalence* (the percentage of a random document representing each possible topic) can be modeled as a function of covariates of the document.

2. *max.em.its* tells **stm()** the maximum number of iterations to use in the EM algorithm before giving up on it ever converging.
3. *init.type="Spectral"* tells stm to initialize the EM algorithm using the spectral decomposition of the term co-occurrence matrix. The spectral initialization uses a deterministic algorithm to find the best starting point for STM's main EM algorithm. According to the team behind Roberts et al. 2019: "In many of the computational and human-judged ways that we'd want to compare models, it tends to produce better models than a random initialization."

4.1 Select K

As discussed in the supplementary, selecting the number of topics **K** is the subject of scientific debate. The code below searches over a provided range of K-values to identify the best-fit, and then assesses provided diagnostic plots to find the best one.

Note this can take a while to run. Skip to the loaded models if easier.

Hide

```
# HCW function; remember to change filenames
K=seq(10,50,by=1)
set.seed(12345)
k_search_hcw <-
  searchK(prepped.hcw_docs$documents,
      prepped.hcw_docs$vocab,
      K = K,
      max.em.its = 75,
      init.type = "Spectral", #important
      prevalence =~ `combined_phq_gad_binarized`,
      data = prepped.hcw_docs$meta,
      verbose=FALSE)
save(k_search_hcw, file="supplementary/searchK-4_hcw_prev.Rdata")
#svg("figs/searchK-3_hcw.svg")
#plot.searchK(k_search_hcw, main="Diagnostic Values by Number of Topics, HCW Cohort")
#dev.off()
```

Hide

```
# Non-HCW function
K=seq(10,50,by=1)
set.seed(12345)
k_search_nonhcw <-
  searchK(prepped.nonhcw_docs$documents,
      prepped.nonhcw_docs$vocab,
      K = K,
      max.em.its = 75,
      init.type = "Spectral", #important
      prevalence =~ `combined_phq_gad_binarized`,
      data = prepped.nonhcw_docs$meta,
      verbose=FALSE)
save(k_search_nonhcw, file="supplementary/searchK-4_nonhcw_prev.Rdata")
#svg("figs/searchK-3_nonhcw.svg")
#plot.searchK(k_search_nonhcw, main="Diagnostic Values by Number of Topics, HCW cohort")
#dev.off()
```

Hide

```
# Modified plot.searchK

custom_plot_searchK<-function(x,plot_title){
  # x: searchK output
  # plot_title <str>: title of the whole plot

  g <- x$results
  oldpar <- par(no.readonly=TRUE)
  #par(mfrow=c(4,1),mar=c(4,4,4,4),oma=c(2,2,2,2))
  par(mfrow=c(4,1),oma=c(0,0,1,0))

  plot(g$K,g$heldout,type="p", xlab="", ylab="Held-Out Likelihood")
  lines(g$K,g$heldout,lty=1,col=1)

  plot(g$K,g$residual,type="p", xlab="", ylab="Residuals")
  lines(g$K,g$residual,lty=1,col=1 )

  if(!is.null(g$semcoh)){
    plot(g$K,g$semcoh,type="p", xlab="", ylab="Semantic Coherence")
    lines(g$K,g$semcoh,lty=1,col=1 )
  }

  plot(g$K,g$exclus,type="n", xlab="Number of Topics (K)", ylab="Exclusivity")
  lines(g$K,g$exclus,lty=1,col=1 )

  #plot(g$K,g$bound,type="n", main="Bound", xlab="Number of Topics (K)", ylab="Bound")
  #lines(g$K,g$bound,lty=1,col=1 )

  #plot(g$K,g$lbound,type="p", main="Lower Bound", xlab="Number of Topics (K)", ylab="Lower Bound")
  #lines(g$K,g$lbound,lty=1,col=1 )

  title(plot_title, outer=TRUE)
  par(oldpar)
}

svg("figs/searchK-4_hcw.svg")
custom_plot_searchK(k_search_hcw, "Statistical Diagnostic Values for HCW Set")
dev.off()

svg("figs/searchK-4_nonhcw.svg")
custom_plot_searchK(k_search_nonhcw, "Statistical Diagnostic Values for Non-HCW Control Set")
dev.off()
```

We selected K=30 based on these tests. For more detail on the statistical and methodological considerations to select the number of topics, see the Supplementary.

4.2 Run the models

Hide

```
K=30

set.seed(12345)
binaryPrevHCW.fit <-
  stm(prepped.hcw_docs$documents,
      prepped.hcw_docs$vocab,
      K = K, # number of topics
      prevalence =~ `combined_phq_gad_binarized`,
      max.em.its = 75,
      data = prepped.hcw_docs$meta,
      init.type = "Spectral",
      verbose=FALSE)
save(binaryPrevHCW.fit, file=paste("binaryPrevHCW_fit.Rdata", sep=""))
```

Hide

```
K=30

set.seed(12345)
binaryPrevControl.fit <-
  stm(prepped.nonhcw_docs$documents,
      prepped.nonhcw_docs$vocab,
      K = K, # number of topics
      prevalence =~ `combined_phq_gad_binarized`,
      #content =~ `omni`,
      max.em.its = 75,
      data = prepped.nonhcw_docs$meta,
      init.type = "Spectral",
      verbose=TRUE)
save(binaryPrevControl.fit, file=paste("binaryPrevControl5_fit.Rdata", sep=""))
```

4.3 Alternatively, start from a loaded model here

Hide

```
K=30
load("data/binaryPrevHCW_fit.Rdata")
load("data/binaryPrevControl5_fit.Rdata")
```

Per the supplementary, we are interested in using semantic coherence and exclusivity to characterize a given model. The below code is an example of how to print those plots two ways.

Hide

```
semCoh <- semanticCoherence(binaryPrevHCW.fit, prepped.hcw_docs$documents, M=10)
exclus <- exclusivity(binaryPrevHCW.fit, M=10, frexw=0.7)
plot(semCoh, exclus,
     xlab="Semantic Coherence", ylab="Exclusivity",
     xlim=c(-40,0), ylim=c(6,10),
     #type="n",
     cex=3, col="red",
     main=paste("Semantic Coherence x Exclusivity (K=",K,")", sep="")
     )
text(semCoh, exclus, labels=c(1:30), cex=0.8, col="red")
```

Hide

```
topicQualMetrics <- topicQuality(binaryPrevHCW.fit, prepped.hcw_docs$documents, main=paste("Semantic Coherence x Exclusivity
(K=",K,")", sep=""))
```

5 Explore the result

The first step in exploring the topic model results is to examine the topics to get a sense of what lexical features in the corpus they might reflect.

5.1 Plot topics by prevalence

Calling **plot.STM()** on the fit object will show us the topics and their relative shares at a glance.

Hide

```
topicProportions <- colSums(binaryPrevHCW.fit$theta, dims=1) / sum(colSums(binaryPrevHCW.fit$theta, dims=1))
topicNames <- list()
for (idx in c(1:K)) {
    topicNames[idx] <- paste("H", idx, " (", round(topicProportions[idx], digits=3), "): ", sep="")
}

plot.STM(binaryPrevHCW.fit,
         topics=1:K,
         labeltype = "frex", n=3,
         topic.names=topicNames,
         main="HCW Topics by Expected Topic Proportions"
         )
```

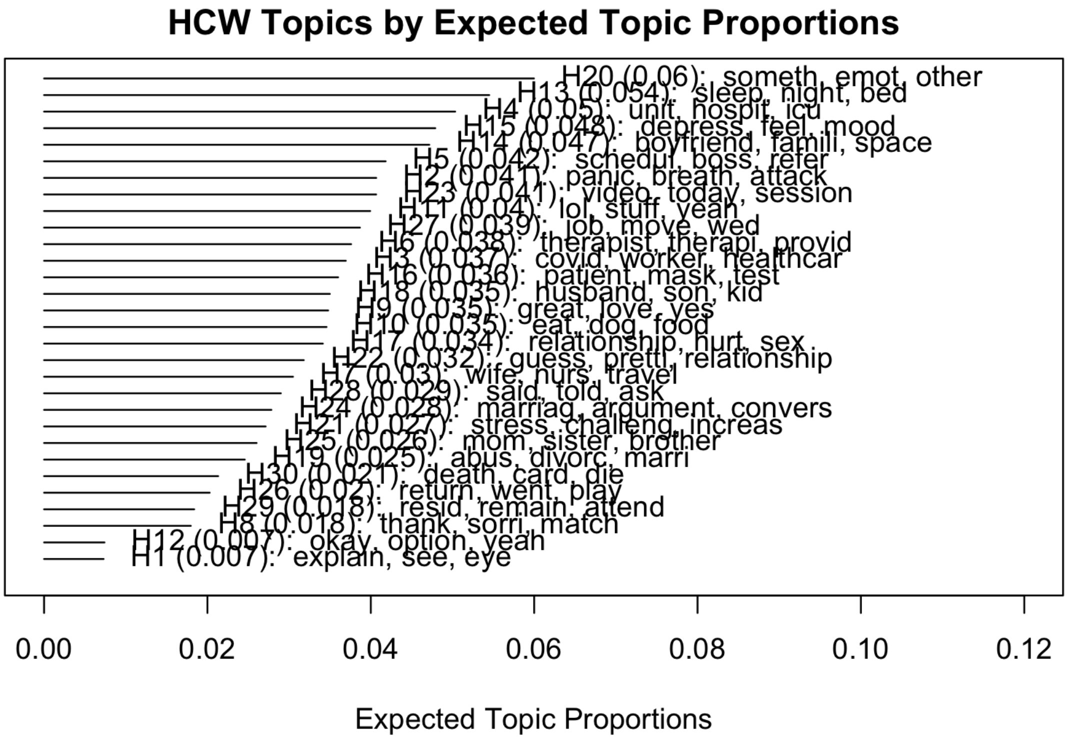

Hide

```
topicProportions <- colSums(binaryPrevControl.fit$theta, dims=1) / sum(colSums(binaryPrevControl.fit$theta, dims=1))
topicNames <- list()
for (idx in c(1:K)) {
    topicNames[idx] <- paste("C", idx, " (", round(topicProportions[idx], digits=3), "): ", sep="")
}

plot.STM(binaryPrevControl.fit,
         topics=1:K,
         labeltype = "frex", n=3,
         topic.names=topicNames,
         main="Control Topics by Expected Topic Proportions"
         )
```

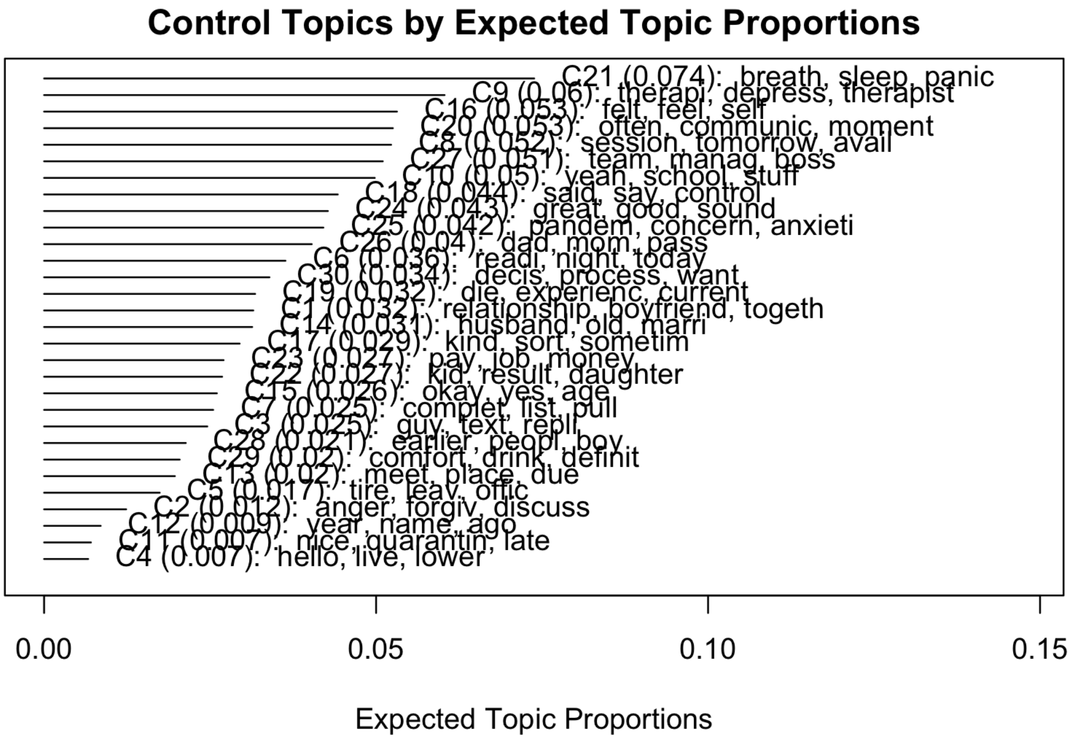

The two code chunks below will save the figures directly your local directory.

5.2 Examine topic labels

The **labelTopics()** function will display the top n words of each topic, using four different metrics: - *prob* ranks words by their raw prevalence in the topic - *frex* takes the harmonic mean of raw prevalence and exclusivity - *score* best words by score metric from the **lda** package - *lift* best words by lift metric from the **textir** package

Each metric produces a slightly different list of words. We truncate here and write to file for cleanliness.

Hide

```
sink('binaryPrevControl5-topics-10.txt')
labelTopics(binaryPrevControl.fit, n = 10)
sink()
```

Hide

```
sink('binaryPrevHCW-topics-10.txt')
labelTopics(binaryPrevHCW.fit, n = 10)
sink()
```

5.3 Examine source documents

It's always good to sanity-check by looking at which documents have been linked with what topics. (Note: we exclude the output here to avoid displaying raw data, but running this cell with data should replicate it.)

Hide

```
doc.text <- prepped.hcw_docs$meta$`txt`
sample.docs <- findThoughts(binaryPrevHCW.fit,
                            texts = doc.text,
                            n = 10,
                            )$docs
sample.docs[[1]]
```

5.4 Compare topic vocabularies

It can also be illustrative to directly compare topics of interest in terms of their vocabulary. This step is to ensure to that what we think are the distinguishing characteristic of each topic do actually distinguish them. The function **plot.STM()** has a plot *type="perspectives"* that serves this purpose. Using this function we can see, for example, that in the HCW set topics 3 and 16 are distinguished by 3 focusing more on COVID and 16 focusing more on patients.

Hide

```
plot.STM(binaryPrevHCW.fit, type="perspectives", topics=c(3,16), n=100, labeltype="frex")
```

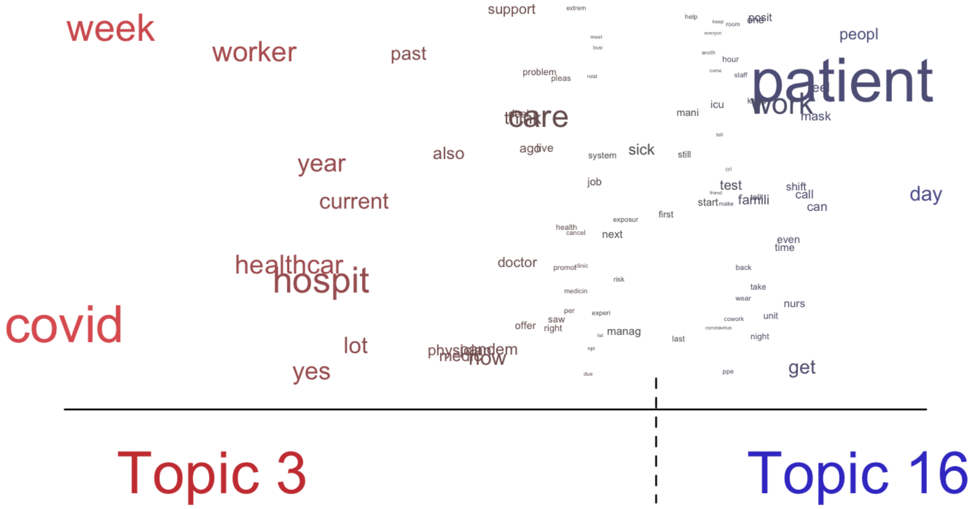

6 Investigate covariate/topic relationships

The package STM also enables us to analyze our mental health outcomes as covariates of topic prevalence.

6.1 Fit effects

The first step is estimate model effects from our fit object. The **estimateEffect()** function takes a formula (we need to specify which topics as the outcome variable), the model fit to predict from, the meta data, and the type of uncertainty estimate to use. The default uncertainty setting, *"Global"*, incorporates topic proportion uncertainties into the displayed uncertainty estimates. We here analyze all identified topics (K=30).

Hide

```
K=30
binaryPrevHCW.effects <- estimateEffect(1:K ~ `combined_phq_gad_binarized`,
                              binaryPrevHCW.fit,
                              documents=prepped.hcw_docs$documents,
                              meta = prepped.hcw_docs$meta,
                              uncertainty = "Global",
                              prior=1e-5)
save(binaryPrevHCW.effects, file="fit_effects_binaryPrevHCW.RData")
```

Hide

```
K=30
binaryPrevControl.effects <- estimateEffect(1:K ~ `combined_phq_gad_binarized`,
                              binaryPrevControl.fit,
                              documents=prepped.nonhcw_docs$documents,
                              meta = prepped.nonhcw_docs$meta,
                              uncertainty = "Global",
                              prior=1e-5)
save(binaryPrevControl.effects, file="fit_effects_binaryPrevControl5.RData")
```

Hide

```
# alternatively, start from loaded effects plots here
load("fit_effects_binaryPrevHCW.RData")
load("fit_effects_binaryPrevControl5.RData")
```

6.2 Plot the estimated topic prevalence differences.

We focus on *"difference"*, a method that plots for each topic the difference in mean prevalence among low vs moderate to severe anxiety/depression cohorts. A mean topic prevalence at the center line at 0.00 indicates the topic is, on average, equally prevalent in both cohorts. When mean topic prevalence is towards the left-hand side of the center line, the topic is more prevalent in low-psychopathology cohorts. When it is towards the right-hand side of the plot, the topic is more prevalent in moderate to severe anxiety/depression cohorts.

Hide

```
topicNames <- list()
topicLabels <- labelTopics(binaryPrevHCW.fit, topics=1:K, n=10)
for (idx in c(1:K)) {
    frexwords <- paste(topicLabels$frex[idx,1], ", ", topicLabels$frex[idx,2], ", ", topicLabels$frex[idx,3], sep="")
    topicNames[idx] <- paste("H", idx, ": ", frexwords, sep="")
}
```

Hide

```
plot.estimateEffect(binaryPrevHCW.effects,
                    covariate = "combined_phq_gad_binarized",
                    model = binaryPrevHCW.fit,
                    method = "difference",
                    cov.value1 = 1, cov.value2 = 0,
                    xlab = "none to mild <--------> moderate to severe",
                    main = "Effect of Anxiety/Depression on Mean Topic Prevalence, HCW set",
                    xlim = c(-.1, .1),
                    labeltype = "frex", # use "frex" to use stm's "frex" to rank top words
                    n = 3, # when non-custom label type, only list the top 3 words
                    custom.labels = topicNames,
                    verbose.labels = F, # labels get spammy with this T
                    )
```

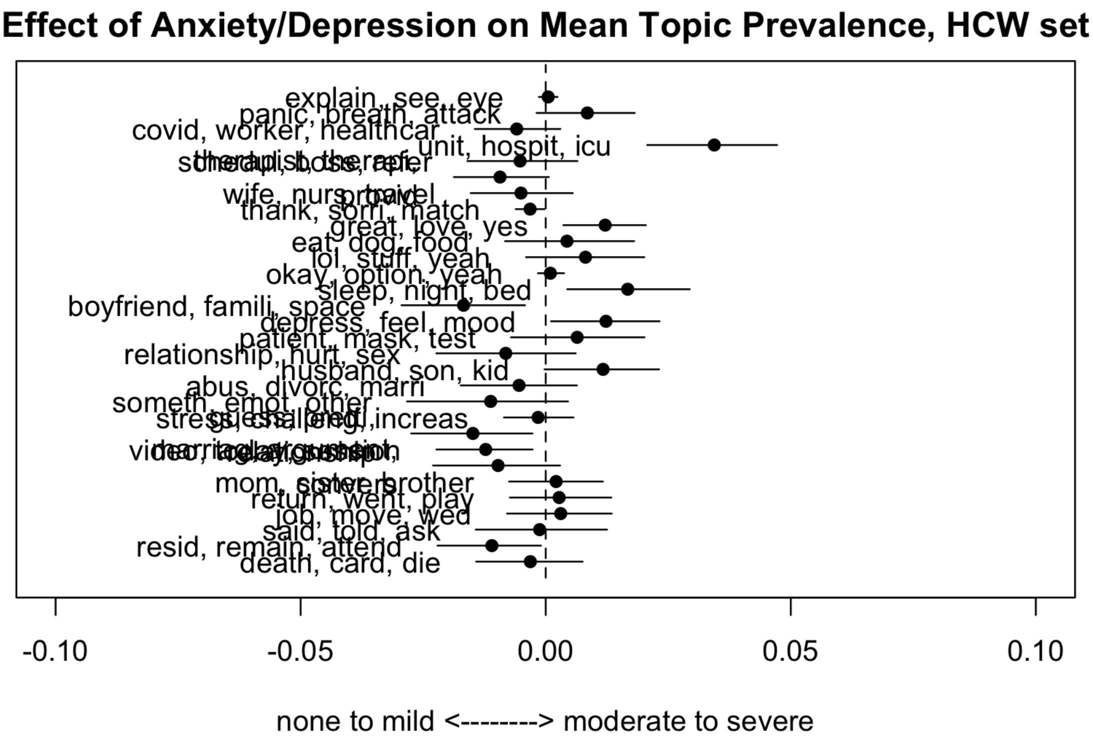

Hide

```
topicNames <- list()
topicLabels <- labelTopics(binaryPrevControl.fit, topics=1:K, n=10)
for (idx in c(1:K)) {
    frexwords <- paste(topicLabels$frex[idx,1], ", ", topicLabels$frex[idx,2], ", ", topicLabels$frex[idx,3], sep="")
    topicNames[idx] <- paste("C", idx, ": ", frexwords, sep="")
}
```

Hide

```
plot.estimateEffect(binaryPrevControl.effects,
                    covariate = "combined_phq_gad_binarized",
                    model = binaryPrevControl.fit,
                    method = "difference",
                    cov.value1 = 1, cov.value2 = 0,
                    xlab = "none to mild <--------> moderate to severe",
                    main = "Effect of Anxiety/Depression on Mean Topic Prevalence, Control Set",
                    xlim = c(-.1, .1),
                    labeltype = "frex", # use stm's "frex" to rank top words
                    n=3, #number of words to display
                    #custom.labels = topicNames,
                    verbose.labels = F, # labels get spammy with this T
                    width = 200,
                    )
```

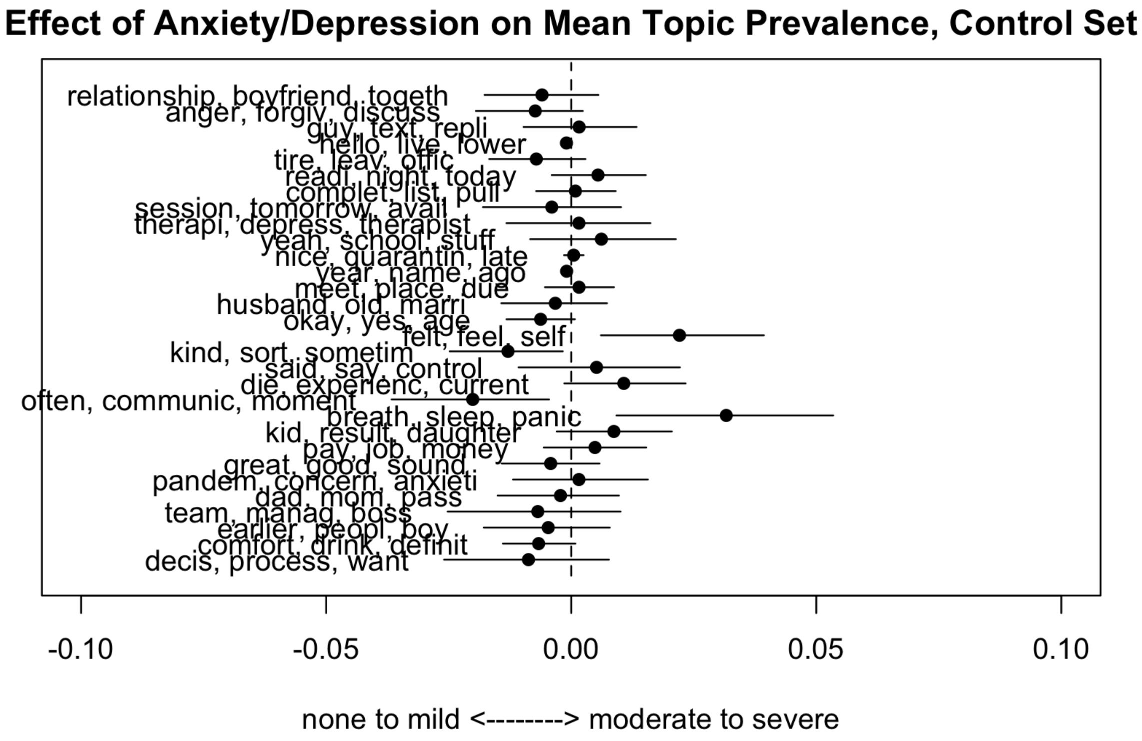

Finally, we can use the summary method of **estimateEffect()** to obtain coefficients estimates for each topic. We truncate them in output here and write to file for cleanliness.

Hide

```
sink('binaryPrevHCW-effects-summary.txt')
summary(binaryPrevHCW.effects)
sink()
```

Hide

```
sink('binaryPrevControl5-effects-summary.txt')
summary(binaryPrevControl.effects)
sink()
```

Based on the numerical estimates, we can now produce a plot with the relevant topics organized by coefficients size:

Hide

```
#combines the following two plots in the same output
# figure sizeing to avoid overlap between labels and dotted line
par(mfrow=c(1,2))  # 1 row, 2 colums for the plots
#labels for quadrants, spacing for accurate position in base r plot
label_box <- "      None to mild symptoms           Moderate to severe symptoms"
#Controls plot
plot.estimateEffect(binaryPrevControl.effects,
                    covariate = "combined_phq_gad_binarized",
                    #ordering topics by estimate size
                    topics =  c(21, 16, 19,25,9,11,27,2),
                    model = binaryPrevControl.fit,
                    method = "difference",
                    cov.value1 = 1, cov.value2 = 0,
                    main = "Controls",
                    xlab = "Estimate",
                    width = 100,
                    xlim = c(-.1, .1),
                    labeltype = "frex",
                    n = 3,
                    custom.labels = topicNames,
                    verbose.labels = F, # labels get spammy with this T,
                    cex.main=1.5
                   )
mtext(side = 3, label_box, line = -1.1, font=2) #adds box labels
```

Hide

```
#HCWs plot
plot.estimateEffect(binaryPrevHCW.effects,
                    covariate = "combined_phq_gad_binarized",
                    #ordering topics by estimate size
                    topics =  c(4,13,15,2,16,21,30,3,29),
                    model = binaryPrevHCW.fit,
                    method = "difference",
                    cov.value1 = 1, cov.value2 = 0,
                    main = "Healthcare Workers",
                    xlab = "Estimate",
                    width = 100,
                    xlim = c(-.1, .1),
                    labeltype = "frex",
                    n = 3,
                    custom.labels = topicNames,
                    verbose.labels = F, # labels get spammy with this T,
                    cex.main=1.5
                   )
mtext(side = 3, label_box, line = -1.1, font=2) #adds box labels
```

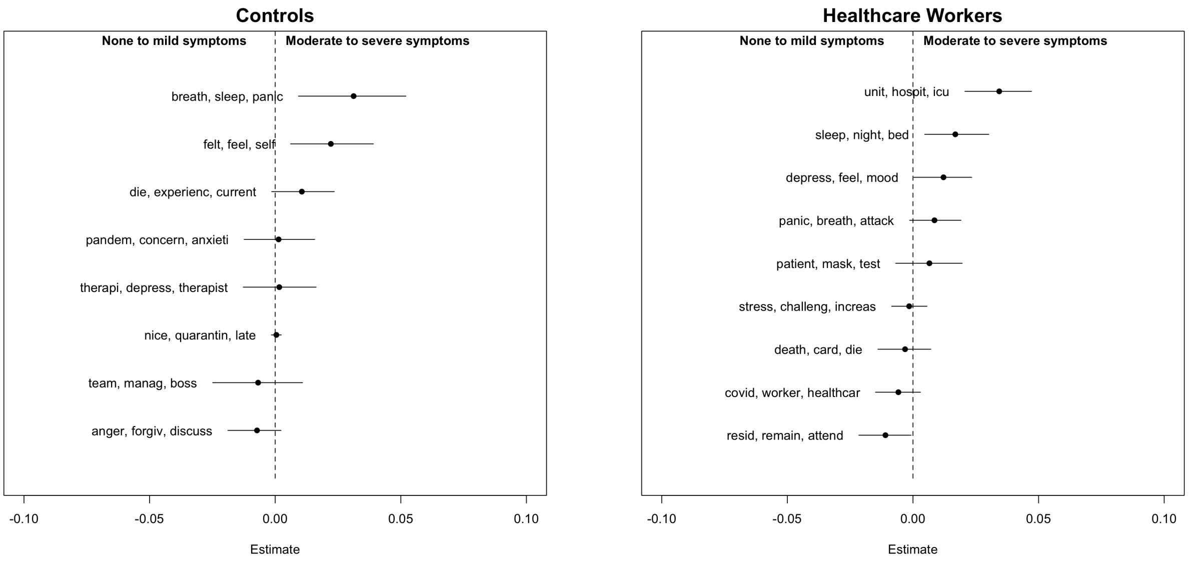

Supplement: Multimedia Appendix 2 [file ai_v2i1e47223_app2.pdf]
