# Supplementary material for: Association of Health Care Work With Anxiety and Depression During the COVID-19 Pandemic: Structural Topic Modeling Study
Source: JMIR AI. 2023 Oct 24;2:e47223. doi: 10.2196/47223 (PMC11041488; doi:10.2196/47223)
Supplement: Multimedia Appendix 3 [file ai_v2i1e47223_app3.pdf]

Heuristic algorithm for identifying HCW jobs

In [1]:
```
import pandas as pd
import numpy as np
import os

from collections import Counter
from collections import defaultdict
```

In [2]:
```
hcws = pd.read_csv("data/hcw-txts-patient-weeks3-phqsonly.csv", index_col=0)
hcws = hcws[['room_id','toks']].reset_index(drop=True)
```

In [3]:
```
roles_to_toks = {
        "med": ["md","doctor","physician","surgeon","cardiologist","anesthesiologist","psychiatrist", \
                "radiologist","dentist", "infectious disease"],
        "medgrad": ["resident","PGY-1","PGY1","PGY-2","PGY2","PGY-3","PGY3","PGY-4","PGY4","residency", \
                    "medical student"],
        "nurse": ["nurse","np","rn","nursing assistant","cna","lvn","APRN","LPN","ACNP","CNS","ANP","FNP", \
                  "GNP","PNP","TCRN", "nurse practitioner","registered nurse", "nursing assistant"],
        "pa": ["pa","clinical officer","physician assistant","physician associate","assistant doctor", \
               "Clinical Liaison"],
        "psych": ["psychologist","psychology intern","psychology fellow","counselor","psyd", "psychology", 'lmhc'],
        "socialworker": ["social worker","LCSW","LMSW", "lsw", "bsw", "msw"],
        "admin": ["administrator", "scribe"],
        "therapist": ["physical therapist","pt","occupational therapist","ot"],
        "ems": ["first responder", "paramedic",],
        "pharm": ["pharmacist", "pharmacy"],
        "lab": ["technologist", "lab tech", "technician", "genetic counselor", "pathologist"],
        # "dentist": ["dentist", "dental"],
        "other": ["dietitian", "nutritionist"],
    }
toks_to_roles = {}
for role,v in roles_to_toks.items():
    for tok in v:
        toks_to_roles[tok.lower()] = role
roles = set(toks_to_roles.values())
roles
```

Out[3]:
```
{'admin',
 'ems',
 'lab',
 'med',
 'medgrad',
 'nurse',
 'other',
 'pa',
 'pharm',
 'psych',
 'socialworker',
 'therapist'}
```

In [4]:
```
def get_all_indices(arr, item):
    """Custom finditer but for arrays not strings bc we are working with tokens
    """
    return [idx for idx, element in enumerate(arr) if element == item]

assert get_all_indices(["hello", "goodbye", "hello"], "hello") == [0,2]
```

In [5]:
```
def find_occupation(toks, mapping, tok_window):
    """ given a mapping of tokens to roles (e.g., "doctor": "md") and some tokenized text,
        identifies whether a role is mentioned in the text.

        Generates a dictionary:
        {
            "decision": decision,
            "mentions": {
                <role>: [keyword, keyword...]
                ...
            },
            "i-ams": {
                <role>: [keyword, keyword...]
                ...
            },
            "snippets": {
                <keyword>: [snippet1, snippet2...]
                ...
            }
        }
    """
    unigrams = toks.split(' ')
    grams = {
        1: unigrams,
        2: [" ".join(unigrams[idx:idx+2]) for idx in range(len(unigrams)-2)],
```

```
    3: [" ".join(unigrams[idx:idx+3]) for idx in range(len(unigrams)-3)],
    4: [" ".join(unigrams[idx:idx+4]) for idx in range(len(unigrams)-4)]
}
unigram_counter = Counter(grams[1])
bigram_counter = Counter(grams[2])

# we will look for whether the role is mentioned after "i am a" or equivalent
being_indicators = {
    1: ["practicing", "being"],
    2: ["being a", "being an", "i practice", "working in", "working as", "my job", "i been", "i an", "i a"],
    3: ["i am a", "i m a", "i am an", "i m an", "i been a", "i work in"],
    4: ["i work as a", "my work as a", "my job as a", "i work as an", "my work as an", "my job as an"],
}
flat_being_indicators = list()
for k,v in being_indicators.items():
    flat_being_indicators.extend(v)

# EXPERIMENT: remove i-ams if they actually begin with these. e.g. "seeing a doctor"
negation_indicators = [
    "seeing a",
    "going to a",
    "started seeing a",
    "been to a",
    "i am not",
]

raw_matches = defaultdict(list)
snippets = defaultdict(list)
being_matches = defaultdict(list)

# first do raw matching
for tok, role in mapping.items():
    if tok in unigram_counter:
        # find the snippet
        indices = get_all_indices(unigrams, tok)
        for idx in indices:
            lidx = idx-tok_window if idx-tok_window >= 0 else 0
            ridx = idx+tok_window if idx+tok_window <= len(unigrams) else len(unigrams)
            snippet = " ".join(unigrams[lidx:ridx])
            snippets[tok].append(snippet)
            antecedent = " ".join(unigrams[lidx:idx])
            # check if your negation indicators are present (like "seeing a")
            negation = False
            for negation_indicator in negation_indicators:
                if negation_indicator in antecedent:
                    negation = True
            # special case for "my doctor" etc
            if antecedent.split(" ")[-1] == "my":
                negation = True
            if not negation:
                # use snippets to add to matches
                for being_indicator in flat_being_indicators:
                    # must be before the mention of the tok
                    if being_indicator in antecedent:
                        being_matches[role].append(tok)
                # add to mentions
                raw_matches[role].append(tok)
    if tok in bigram_counter:
        # add to mentions
        raw_matches[role].append(tok)
        # add to snippets
        indices = get_all_indices(grams[2], tok)
        for idx in indices:
            lidx = idx-tok_window if idx-tok_window >= 0 else 0
            ridx = idx+tok_window if idx+tok_window <= len(unigrams) else len(unigrams)
            snippet = " ".join([bigram.split(" ")[0] for bigram in grams[2][lidx:ridx]])
            snippets[tok].append(snippet)
            antecedent = " ".join([bigram.split(" ")[0] for bigram in grams[2][lidx:idx]])
            # check if your negation indicators are present (like "seeing a")
            negation = False
            for negation_indicator in negation_indicators:
                if negation_indicator in antecedent:
                    negation = True
            # special case for "my doctor" etc
            if antecedent.split(" ")[-1] == "my":
                negation = True
            if not negation:
                # use snippets to add to matches
                for being_indicator in flat_being_indicators:
                    # must be before the mention of the tok
                    if being_indicator in antecedent:
                        being_matches[role].append(tok)
                # add to mentions
                raw_matches[role].append(tok)

# if either heuristic surfaces a single role, go with it. otherwise call it an UNK.
```

```python
    # if someone says they're a "physician assistant" you have to hard-code (also catches med)
    if 'pa' in being_matches:
        decision = "pa"
    # if someone says they're a resident physician, they show up as both a resident and physician.
    elif set(being_matches.keys()) == set(["medgrad", "med"]):
        decision = 'medgrad'
    elif len(being_matches) == 1:
        decision = list(being_matches.keys())[0]
    # if you have no i-ams and only mentions, and the only mention is a psych practitioner, skip
    elif (len(being_matches) == 0 and len(raw_matches) == 1):
        role = list(raw_matches.keys())[0]
        tok = list(raw_matches.values())[0][0]
        if tok == "psychiatrist" or role == "psych":
            decision = "UNK"
        else:
            decision = role
    elif len(raw_matches) == 1:
        decision = list(raw_matches.keys())[0]
    elif len(being_matches) == 0 and len(raw_matches) == 0:
        decision = "NO-MENTION"
    else:
        decision = "UNK"

    return {
        "decision": decision,
        "mentions": raw_matches,
        "i-ams": being_matches,
        "snippets": snippets,
    }

# find_occupation(hcws.iloc[763]['toks'], toks_to_roles, 5)
```

In [6]:

```python
def generate(ilocs, dataset, toks_to_roles, tok_window):
    """ Given a set of ilocs and a dataset (HCW or control), runs the find_occupation algorithm for each iloc.
        Returns:
            outs <dict dict>: all output for each datapoint, e.g.
                { <iloc>: {
                    'decision': 'nurse',
                    'mentions': defaultdict(list,
                        {'nurse': ['nurse', 'rn', 'nursing', 'registered nurse']}),
                    'i-ams': defaultdict(list,
                        {'nurse': ['rn', 'registered nurse']})
                    },
                  ...,
                }
            Y_hat <dict str>: just the decisions for each datapoint: { <iloc>: 'nurse', ... }
            failures <list int>: list of indices where it errored
    """
    outs = dict()
    Y_hat = dict()
    failures = list()
    for iloc in ilocs:
        try:
            outs[iloc] = find_occupation(dataset.iloc[iloc]['toks'], toks_to_roles, tok_window)
            Y_hat[iloc] = outs[iloc]['decision']
        except KeyboardInterrupt:
            return
        except:
            failures.append(iloc)
    print('failed {}/{} samples'.format(len(failures), len(ilocs)))
    return outs, Y_hat, failures

outs, Y_hat, failures = generate(range(0,10), hcws, toks_to_roles, 5)
```

```
failed 0/10 samples
```

Initial development

To get a seed set of labeled data, we'll start with smaller labeled sets of the first 10 HCWs, then the first 30.

Start with n=10 labels

In [7]:

```python
# hand-labeling the first 10 HCWs
Y = {
    0: 'nurse', # works with raw-match and i-am
    1: 'med', # correct answer present, needs disambiguation
    2: 'nurse', # works with i-am
    3: 'therapist', # works with raw-match
    4: 'UNK', # incorrectly identifies a role
    5: 'ems', # works with raw-match
    6: 'med', # correct answer present, needs disambiguation
    7: 'UNK', # incorrectly identifies a role
    8: 'nurse', # works with i-am
```

```
    9: 'nurse', # works with i-am
}
```

In [8]:
```
outs, Y_hat, failures = generate(range(0,10), hcws, toks_to_roles, 5)
```

```
failed 0/10 samples
```

In [9]:
```
from sklearn import metrics
import matplotlib.pyplot as plt

def compare(ground_truth, generated, mapping):
    """ Takes in Y, Y_hat, and the mapping (w/ the expected categories).
        Tabulates what the algo got right and wrong.
        Returns:
            metrics <dict>:
            cm <np arr (n_col, n_col)> where n_col is the number of distinct roles derived from the mapping
    """
    assert ground_truth.keys() == generated.keys() # make sure indices line up
    # builds confusion matrix
    cols = list(mapping.keys())
    cols.extend(["NO-MENTION", "UNK"])
    cols.sort()
    n_col = len(cols)
    cm = np.zeros((n_col, n_col))
    # tallies various relevant metrics
    counts = {
        "correct_hcw": list(),
        "incorrect_hcw": list(),
        "false_pos": list(),
        "false_neg": list(),
        "true_neg": list(),
    }
    non_hcws = set(["UNK", "NO-MENTION"])
    for iloc in list(ground_truth.keys()):
        y = ground_truth[iloc]
        y_hat = generated[iloc]
        cm[cols.index(y), cols.index(y_hat)] += 1
        if y in non_hcws:
            if y_hat in non_hcws:
                counts["true_neg"].append(iloc)
            else:
                counts["false_pos"].append(iloc)
        else:
            if y_hat in non_hcws:
                counts["false_neg"].append(iloc)
            elif y == y_hat:
                counts["correct_hcw"].append(iloc)
            else:
                counts["incorrect_hcw"].append(iloc)

    # HCW v nonHCW correct rate
    all_correct = len(counts["correct_hcw"]) + len(counts["incorrect_hcw"]) + len(counts["true_neg"])
    print("{}/{} or {:%} HCW v nonHCW correct (correct_hcw + incorrect_hcw + true_neg)" \
          .format(all_correct, len(ground_truth),all_correct/len(ground_truth)))


    # Correct rate
    precise_correct = len(counts["correct_hcw"]) + len(counts["true_neg"])
    print("{}/{} or {:%} precise correct (correct_hcw + true_neg)".format(precise_correct, len(ground_truth), \
                                                                          precise_correct/len(ground_truth)))
    #
    all_hcw = len(counts["correct_hcw"]) + len(counts["incorrect_hcw"])
    print("{}/{} or {:%} hcw correct (correct_hcw / all hcw)".format(len(counts["correct_hcw"]), all_hcw, \
                                                                     len(counts["correct_hcw"])/all_hcw))
    # FPR = FP / N = FP / (FP + TN)
    num_neg = len(counts["false_pos"]) + len(counts["true_neg"])
    if num_neg > 0:
        print("{}/{} or {:%} fpr (proportion of pos false)".format(len(counts["false_pos"]), num_neg, \
                                                                   len(counts["false_pos"])/num_neg))
    # FNR = FN / P = FN / (FN + TP)
    num_pos = len(counts["false_neg"]) + len(counts["correct_hcw"]) + len(counts["incorrect_hcw"])
    if num_pos > 0:
        print("{}/{} or {:%} fnr (proportion of HCWs)".format(len(counts["false_neg"]), \
                                                              num_pos, len(counts["false_neg"])/num_pos))

    # Print the confusion matrix
    confusion_matrix = metrics.confusion_matrix(
        y_true = list(ground_truth.values()),
        y_pred = list(generated.values()),
        labels = cols,
        normalize='true',
    )
    cm_display = metrics.ConfusionMatrixDisplay(confusion_matrix,
        display_labels=cols,
    )
    cm_display.plot(xticks_rotation='vertical')
```

```python
    # plt.show()
    plt.savefig("figs/hcw_162sample_cm.png")

    return counts, cm

counts, cm = compare(Y, Y_hat, roles_to_toks)
```

```
10/10 or 100.000000% HCW v nonHCW correct (correct_hcw + incorrect_hcw + true_neg)
9/10 or 90.000000% precise correct (correct_hcw + true_neg)
7/8 or 87.500000% hcw correct (correct_hcw / all hcw)
0/2 or 0.000000% fpr (proportion of pos false)
0/8 or 0.000000% fnr (proportion of HCWs)
```

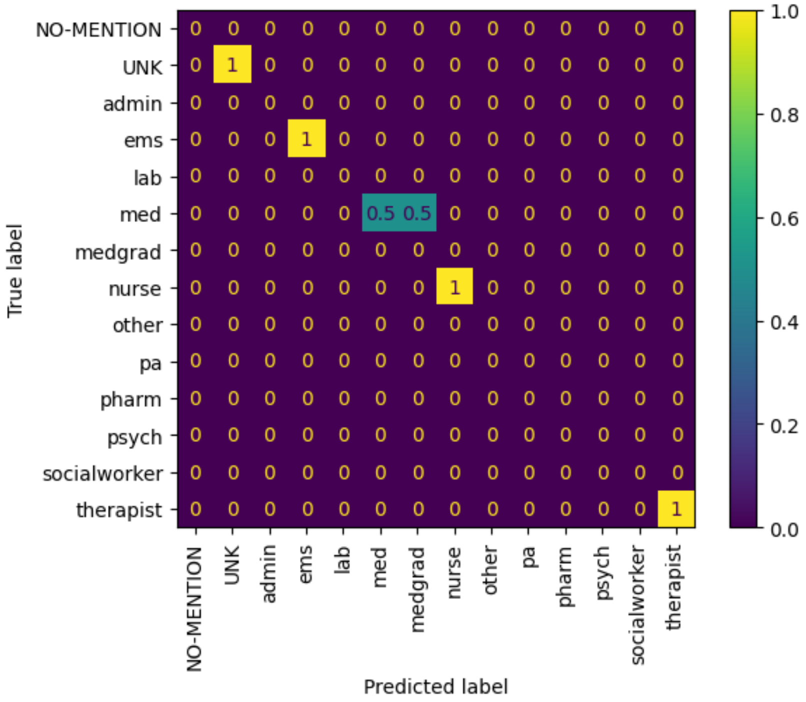

Expand to n=30

In [10]:

```python
# hand-labeling the first N HCWs
Y = {
    0: 'nurse', # works with raw-match and i-am
    1: 'medgrad', # in residency
    2: 'nurse', # works with i-am
    3: 'therapist', # works with raw-match
    4: 'UNK', # incorrectly identifies a role
    5: 'ems', # works with raw-match
    6: 'med', # correct answer present, needs disambiguation
    7: 'NO-MENTION', # incorrectly identifies a role
    8: 'nurse', # works with i-am
    9: 'nurse', # works with i-am,
    10: 'nurse',
    11: 'therapist', # physical therapist assistant
    12: 'nurse',
    13: 'socialworker',
    14: 'nurse',
    15: 'nurse',
    16: 'nurse',
    17: 'nurse',
    18: 'nurse',
    19: 'therapist',
    20: 'NO-MENTION',
    21: 'med',
    22: 'medgrad',
    23: 'pharm',
    24: 'nurse',
    25: 'nurse',
    26: 'nurse',
    27: 'psych',
    28: 'pa',
    29: 'psych',
    30: 'med'
}
```

In [11]:

```python
Counter(Y.values()).most_common()
```

Out[11]:

```
[('nurse', 14),
 ('therapist', 3),
```

```
 ('med', 3),
 ('medgrad', 2),
 ('NO-MENTION', 2),
 ('psych', 2),
 ('UNK', 1),
 ('ems', 1),
 ('socialworker', 1),
 ('pharm', 1),
 ('pa', 1)]
```

In [12]:
```
outs, Y_hat, failures = generate(range(0,31), hcws, toks_to_roles, 5)
counts, cm = compare(Y, Y_hat, roles_to_toks)
```

```
failed 0/31 samples
30/31 or 96.774194% HCW v nonHCW correct (correct_hcw + incorrect_hcw + true_neg)
30/31 or 96.774194% precise correct (correct_hcw + true_neg)
27/27 or 100.000000% hcw correct (correct_hcw / all hcw)
0/3 or 0.000000% fpr (proportion of pos false)
1/28 or 3.571429% fnr (proportion of HCWs)
```

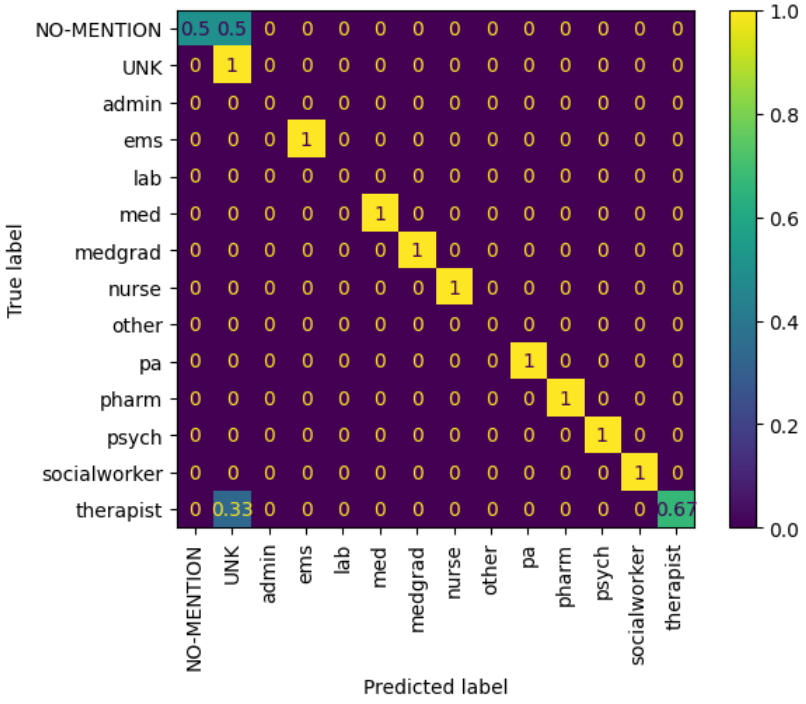

In [13]:
```
def inspect_failures(fail_cases, dataset):
    for fail in fail_cases:
        print("\niloc={}\n=====\n{}".format(fail, dataset.iloc[fail]['toks']))

#inspect_failures(failures[0], hcws)
```

Format data for labeling

To improve our algorithm further, we'll draw larger snippets of data for labeling.

In [14]:
```
def format_snippets(df, generate_out, Y_hat, predict=False):
    """
        df: hcws/control or sample
        generate_out: output of generate
        predict <bool>: whether to include the outputs of the current algo
    """
    output_df = df[["room_id","toks"]]
    snippets_to_label = list()
    for idx,row in output_df.iterrows():
        s = list()
        for snippet_arr in generate_out[idx]["snippets"].values():
            if len(snippet_arr) > 0:
                s.extend(snippet_arr)
        if len(s) == 0:
            snippets_to_label.append(row["toks"])
        else:
            snippets_to_label.append(s)
    output_df["snippets"] = snippets_to_label
    output_df = output_df[["room_id","snippets"]]
    if predict:
        output_df["predictions"] = list(Y_hat.values())
    return output_df

sample = hcws.sample(164)
```

```
outs, Y_hat, failures = generate(sample.index, hcws, toks_to_roles, tok_window=5)
format_snippets(sample, outs, Y_hat, predict=True).to_csv("data/HCW-job_sample_2022-09-02.csv")
```

```
failed 0/164 samples
```

In [15]:
```
# Get UNKs
outs, Y_hat, failures = generate(hcws.index, hcws, toks_to_roles, tok_window=5)
hcws['Y_hat'] = Y_hat.values()
unk_df = hcws[hcws["Y_hat"] == "UNK"]
print(len(unk_df))
unk_outs = {k:v for k, v in outs.items() if k in set(unk_df.index)}
unk_Y_hats = {k:v for k, v in Y_hat.items() if k in set(unk_df.index)}
format_snippets(unk_df, unk_outs, unk_Y_hats, predict=True).to_csv("data/HCW-job_UNKS_2022-09-02.csv")
```

```
failed 0/820 samples
72
```

In [16]:
```
# Get whole set
outs, Y_hat, failures = generate(range(0,len(hcws)), hcws, toks_to_roles, 5)
format_snippets(hcws, outs, Y_hat, predict=True).to_csv("data/HCW-job_all_2022-09-06.csv")
```

```
failed 0/820 samples
```

Expand to UNKs

In [17]:
```
unk_set = pd.read_csv("data/MMlabels_UNKS_2022-08-27.csv", index_col="idx")[["ground_truth"]]
Y = { int(idx): row["ground_truth"] for idx, row in unk_set.iterrows() }
outs, Y_hat, failures = generate(list(unk_set.index), hcws, toks_to_roles, 5)
counts, cm = compare(Y, Y_hat, roles_to_toks)
```

```
failed 0/59 samples
24/59 or 40.677966% HCW v nonHCW correct (correct_hcw + incorrect_hcw + true_neg)
24/59 or 40.677966% precise correct (correct_hcw + true_neg)
10/10 or 100.000000% hcw correct (correct_hcw / all hcw)
1/15 or 6.666667% fpr (proportion of pos false)
34/44 or 77.272727% fnr (proportion of HCWs)
```

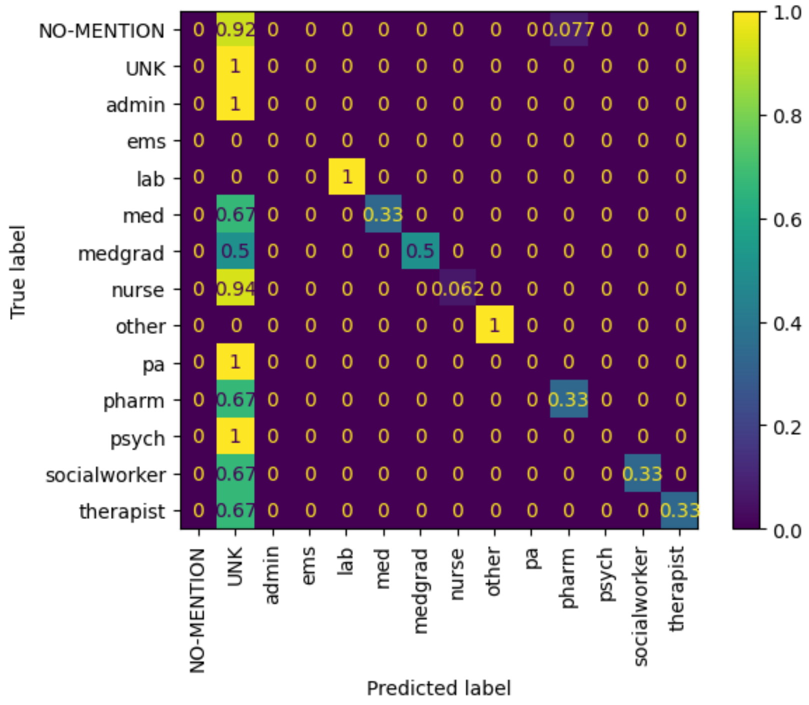

In [18]:
```
def inspect(metric_flag, counts, dataset, Y_hat, Y, mapping, tok_window):
    print("Inspecting all in category: {}".format(metric_flag))
    for idx in counts[metric_flag]:
        print("\niloc={}\n=====".format(idx))
        print("PREDICTED: {}\tGROUND TRUTH: {}".format(Y_hat[idx], Y[idx]))
        decision_output = find_occupation(dataset.iloc[idx]["toks"], mapping, tok_window)
        print("\nSNIPPETS:\t{}".format(decision_output["snippets"]))
        print("I-AMS:\t{}".format(decision_output["i-ams"]))
        print("MENTIONS:\t{}".format(decision_output["mentions"]))

#inspect("incorrect_hcw", counts, hcws, Y_hat, Y, toks_to_roles, 5)
```

In [19]:
```
#inspect("false_pos", counts, hcws, Y_hat, Y, toks_to_roles, 5)
```

In [20]:
```
#inspect("false_neg", counts, hcws, Y_hat, Y, toks_to_roles, 5)
```

Above code is used to examine the UNKs and come up with additional heuristic rules, commented out for publication due to privacy concerns exposing

the raw data.

Test the algorithm

We will use as our test set a hand-labeled random selection of 162 HCW samples.

In [21]:
```
test_set = pd.read_csv("data/MMlabels_random_2022-08-27.csv", index_col="idx")[["ground_truth"]]
labels = list(roles_to_toks.keys())
labels.extend(["NO-MENTION", "UNK"])
Y = { int(idx): row["ground_truth"] for idx, row in test_set.iterrows() if row["ground_truth"] in labels}
```

In [22]:
```
outs, Y_hat, failures = generate(list(Y.keys()), hcws, toks_to_roles, 5)
counts, cm = compare(Y, Y_hat, roles_to_toks)
```

```
failed 0/162 samples
134/162 or 82.716049% HCW v nonHCW correct (correct_hcw + incorrect_hcw + true_neg)
128/162 or 79.012346% precise correct (correct_hcw + true_neg)
117/123 or 95.121951% hcw correct (correct_hcw / all hcw)
5/16 or 31.250000% fpr (proportion of pos false)
23/146 or 15.753425% fnr (proportion of HCWs)
```

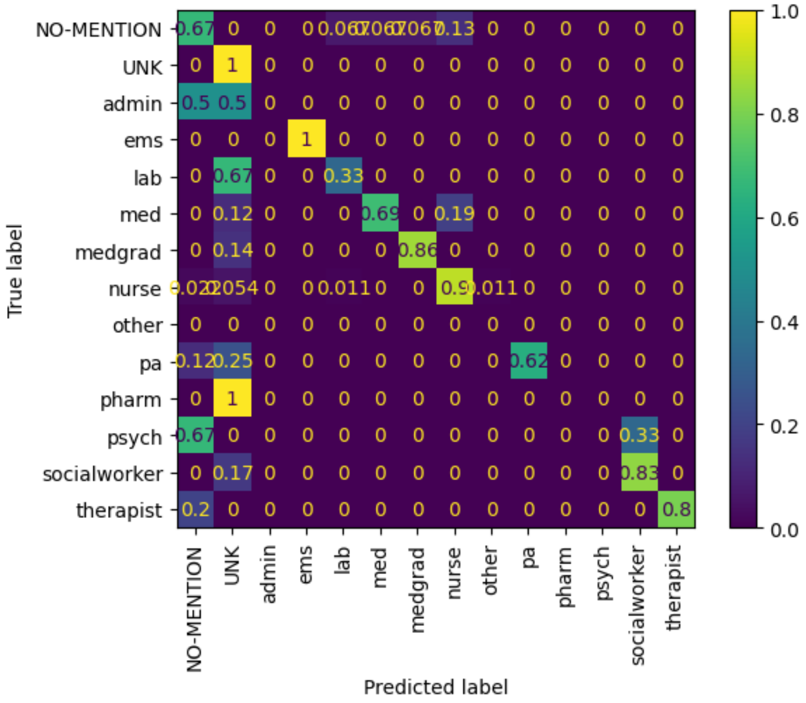

In [23]:
```
# Get the raw counts for Supplementary table
for k,v in counts.items():
    print(k, len(v))
```

```
correct_hcw 117
incorrect_hcw 6
false_pos 5
false_neg 23
true_neg 11
```

In [24]:
```
#inspect("false_neg", counts, hcws, Y_hat, Y, toks_to_roles, 5)
```

Run the algo on the entire HCW set

Now we run the algorithm on the full HCW set to get final estimates.

In [25]:
```
outs, Y_hat, failures = generate(range(0,len(hcws)), hcws, toks_to_roles, 5)
```

```
failed 0/820 samples
```

In [26]:
```
Counter(Y_hat.values()).most_common()
```

Out[26]:
```
[('nurse', 400),
 ('NO-MENTION', 89),
 ('med', 85),
 ('UNK', 72),
 ('medgrad', 50),
 ('therapist', 33),
 ('socialworker', 26),
 ('pa', 20),
 ('pharm', 13),
```

```
 ('lab', 12),
 ('ems', 8),
 ('psych', 8),
 ('other', 3),
 ('admin', 1)]
```

In [27]:
```
def plot_yhats(Y_hat_dict):
    yhats = Counter(Y_hat_dict.values()).most_common()
    yhats_data = [tup[1] for tup in yhats]
    yhats_labels = [tup[0] for tup in yhats]
    # Creating autocpt arguments
    def func(pct, allvalues):
        absolute = int(pct / 100.*np.sum(allvalues))
        return "{:.1f}%\n({:d})".format(pct, absolute)

    fig, ax = plt.subplots(figsize =(10, 7))
    wedges, texts, autotexts = plt.pie(yhats_data, labels=yhats_labels,
        autopct = lambda pct: func(pct, yhats_data),
    )

plot_yhats(Y_hat)
plt.savefig("figs/hcw_820_roles.png")
```

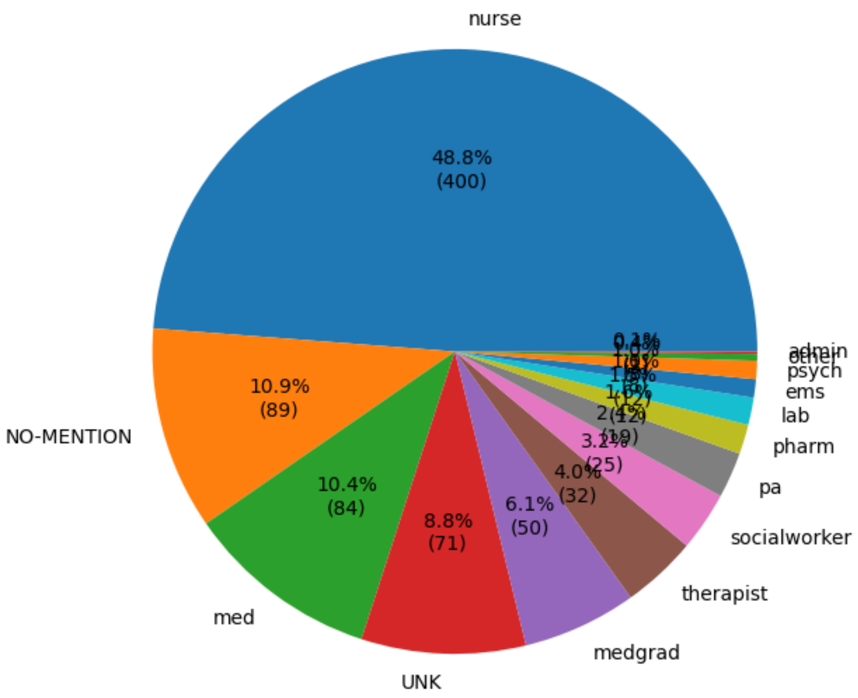

In [28]:
```
#inspect_failures(failures, hcws)
```

What about the control set?

To make sure our job algo works reasonably well, we'll try it on the control set too. We shouldn't get *too many* HCWs here. We can expect some to appear due to the natural errors that come with a heuristic like this, but we can then hand-label them.

In [29]:
```
controls = pd.read_csv("data/HCWcontrols_2022-09-05.csv", index_col=0)
controls = controls.reset_index(drop=True)
```

In [30]:
```
outs, Y_hat, failures = generate(controls.index, controls, toks_to_roles, 5)
format_snippets(controls, outs, Y_hat, predict=True).to_csv("data/Controls-job_all_2022-09-05.csv")
```

```
failed 0/820 samples
/var/folders/h9/_z_8w_zs5js_dqm38pkdt7y40000gn/T/ipykernel_96846/265100142.py:18: SettingWithCopyWarning:
A value is trying to be set on a copy of a slice from a DataFrame.
Try using .loc[row_indexer,col_indexer] = value instead

See the caveats in the documentation: https://pandas.pydata.org/pandas-docs/stable/user_guide/indexing.html#returning-a-view-versus-a-copy
  output_df["snippets"] = snippets_to_label
```

In [31]:
```
Counter(Y_hat.values()).most_common()
```

Out[31]:
```
[('NO-MENTION', 561),
 ('UNK', 127),
```

```
 ('med', 85),
 ('nurse', 18),
 ('therapist', 13),
 ('pharm', 3),
 ('admin', 3),
 ('psych', 3),
 ('lab', 3),
 ('socialworker', 2),
 ('pa', 1),
 ('other', 1)]
```

In [32]:
```
plot_yhats(Y_hat)
```

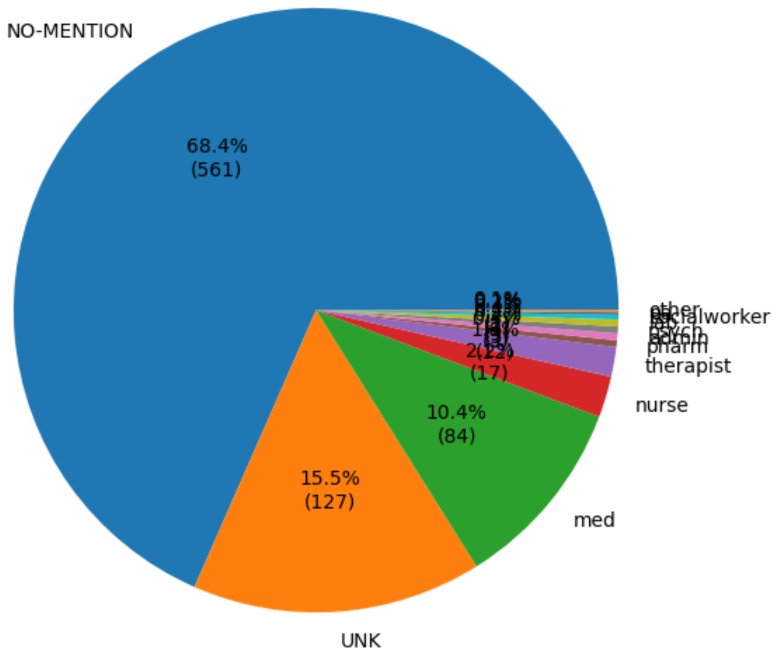

In [33]:
```
Counter(Y_hat.values())['NO-MENTION'] + Counter(Y_hat.values())['UNK']
```

Out[33]:
```
688
```

In [34]:
```
print("{:%} of controls appear as HCWs".format((len(controls) - (Counter(Y_hat.values())['NO-MENTION'] \
                                                        + Counter(Y_hat.values())['UNK']))/len(controls)))
```

```
16.097561% of controls appear as HCWs
```

We were then able to manually inspect the 16.10% of controls that appeared as HCWs. Our review showed they were all erroneous (e.g., someone mentioning their surgeon and getting labeled as a surgeon.) See the Supplementary for more.

In [ ]:
```
```
